# Effects of Future Climate Extreme Heat Events and Land Use Changes on Land Vertebrates

**DOI:** 10.1111/gcb.70625

**Published:** 2025-12-09

**Authors:** Reut Vardi, Gopal Murali, Gabriel Henrique de Oliveira Caetano, Uri Roll, Shai Meiri

**Affiliations:** ^1^ School of Zoology Tel Aviv University Tel Aviv Israel; ^2^ School of Geography and the Environment University of Oxford Oxford UK; ^3^ Department of Ecology and Evolutionary Biology University of Arizona Tucson Arizona USA; ^4^ Centre for Ecological Sciences Indian Institute of Science Bengaluru India; ^5^ Jacob Blaustein Center for Scientific Cooperation, The Jacob Blaustein Institutes for Desert Research Ben‐Gurion University of the Negev, Midreshet Ben‐Gurion Beer‐Sheva Israel; ^6^ Mitrani Department of Desert Ecology, the Jacob Blaustein Institutes of Desert Research Ben‐Gurion University of the Negev, Midreshet Ben‐Gurion Beer‐Sheva Israel; ^7^ School of Biosciences The University of Melbourne Parkville Victoria Australia

**Keywords:** biodiversity, climate change, conservation policy, representative concentration pathway (RCP), shared socioeconomic pathways (SSP)

## Abstract

Climate and land use changes pose the greatest threats to biodiversity, but their synergistic effects on biodiversity have been rarely studied. Here, we assessed the combined effects of future climate and land use changes on the world's land vertebrates (29,657 species) across four socioeconomic scenarios between 2015 and 2100. We evaluated species range exposures to extreme heat events (relative to temperatures during 1950–2005) and land use changes in species' preferred habitats. Under the best‐case scenario, species will experience unsuitable conditions due to both factors across 10% of their range on average. In the worst scenario, species are projected to face unsuitable conditions across 52% of their range. By the end of the century, up to 7895 species are expected to face extreme heat events and/or unsuitable land use changes across their entire range, and thus potentially go globally extinct. The synergistic impact of climate and land use changes is most noticeable in the Sahel (e.g., Sudan, Chad and Mali), the Middle East (e.g., Afghanistan, Iraq and Saudi Arabia), and Brazil. Under two of the four scenarios, over half of all Data Deficient (> 77%), Near Threatened (> 50%), or threatened species (> 60%), will experience unsuitable conditions across at least half of their range. Our results highlight the potential detrimental effects of future environmental changes and the importance of considering and mitigating the synergetic effects of these threats on biodiversity.

## Graphical abstract

Our study demonstrates how the combined effects of extreme heat events resulting from climate change and land‐use change may impact land vertebrates worldwide by 2100. Using global projections across four socioeconomic scenarios, we found that even under the best conditions, species could lose suitable habitat across 10% of their range, rising to over 50% in the worst case, with up to 7895 species facing extinction as a result. Our findings emphasise the urgent need to address both climate and land use pressures together to protect biodiversity.

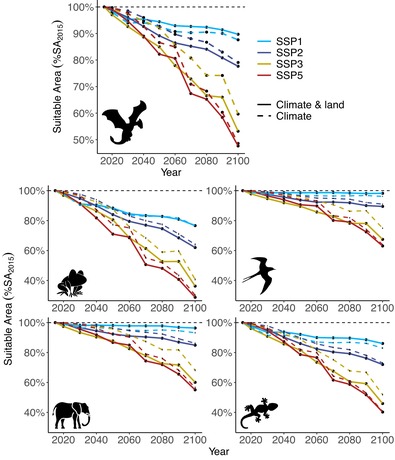

## 1 Introduction

Global biodiversity faces unprecedented challenges from human‐induced environmental changes, with climate change and land use changes being the two most significant global threats (Redlich et al. 2022). While climate and land use changes have been extensively studied individually (Betts et al. 2019), their interactive and synergistic effects on biodiversity remain poorly studied (but see Albaladejo‐Robles et al. 2023; Prieto‐Torres et al. 2022; Williams et al. 2022), leaving a critical knowledge gap in conservation science. Recent studies have highlighted the importance of considering multiple stressors in biodiversity assessments, as their combined effect may differ from the sum of their individual impacts (Montràs‐Janer et al. 2024; Turschwell et al. 2022). For example, land use change can fragment habitats and limit species' ability to disperse or adapt to changing climates (Chan et al. 2024; Guo et al. 2018). Similarly, climate change, particularly extreme heat events, can further compound the risks for species confined to already degraded or unsuitable landscapes (Kotz et al. 2025; Lauck et al. 2023; Segan et al. 2016).

Despite this, global‐scale analyses of such projected interactions remain rare. Outhwaite et al. (2022) showed that the synergistic interactions between increasing temperatures and recent past land use changes caused greater reductions in insect richness and abundance than each factor on its own, especially in the tropics. Such synergetic effects are expected to increase, with some studies suggesting that 49% of remaining wilderness areas will be negatively affected by one or both factors by 2050 (Asamoah et al. 2022), and local species richness will be reduced by 20% by 2070 in regions across the globe (Newbold 2018). Furthermore, extreme heat events are expected to intensify in the future in frequency, duration, and severity and can pose major threats to biodiversity globally (Murali et al. 2023). Such short‐term extremes may have even greater impacts on species than changes in temperature averages (Harris et al. 2018; Murali et al. 2023; Vasseur et al. 2014).

Here, we provide the first comprehensive global assessment of the combined effects of future extreme heat events and land use changes on terrestrial vertebrates. We highlight spatial and taxonomic patterns of these synergistic threats by analysing potential exposure to unsuitable land use and extreme heat events for 29,657 vertebrate species (6407 amphibians, 9472 birds, 5161 mammals, and 8617 reptiles) across four Shared Socioeconomic Pathways (SSP), which address trends in population growth, economic development, energy use, and land use changes spanning 2015–2100. Each SSP was combined with its respective Representative Concentration Pathway (RCP) that epitomises trajectories of greenhouse gas concentrations: SSP1‐RCP2.6, SSP2‐RCP4.5, SSP3‐RCP7.0 and SSP5‐RCP8.5. For climate, we included explicit considerations of extreme heat events per species' thermal maximum evaluated as projected future intensity, duration or frequency higher of extreme heat events compared to species' exposure to such events during 1950–2005.

Our main goal was to estimate the potential loss of suitable habitat due to future land use changes and extreme heat events across current known ranges, as well as to highlight areas of potential suitability to inform conservation policy. Thus, we calculated the proportion of area that will be exposed to unsuitable conditions due to land use changes, extreme heat events, or both, across terrestrial vertebrates' known distributional ranges, by 2100. Species experiencing such extreme climates and losing their preferred habitats are likely to face a higher extinction risk (Britnell et al. 2023). Thus, it is crucial to identify species and areas expected to be the most impacted, and guide conservation plans aimed at minimising biodiversity loss (Cardillo et al. 2023), as well as more favourable areas (Mi et al. 2023).

## 2 Methods

We quantified exposure to extreme heat events and unsuitable habitat types due to land use changes across species' current known ranges. Suitable habitat types per species were obtained from the IUCN (i.e., habitat classification scheme), and future habitat type maps were obtained from the Land‐Use Harmonisation 2 (LUH2) dataset for four different future scenarios (SSP‐RCPs). Extreme heat events data were obtained from Murali et al. (2023). Current range maps were overlapped with the 2015 projection of land use, and only areas under suitable habitat types were considered as suitable for 2015. By overlapping current range maps with future land use (LUH2) and extreme heat events (2020–2100), we estimate future suitable and unsuitable areas for each species. We calculated the proportional change in the suitable area for each species, with its 2015 suitable area serving as 100% of the area it occupies. Overall, we analyzed species' suitable habitats at a resolution of 24.125 × 24.125 km^2^ (see details below). Consequently, we did not introduce potential effects of connectivity and dispersal in our analyses (see further details below) because we aimed to provide a maximum assessment of potential suitability within current ranges.

### 2.1 Species Range Maps and Datasets

We obtained species range maps from the IUCN (iucnredlist.org/; version 6.2) for amphibians and mammals, from BirdLife for birds (http://datazone.birdlife.org/; version 4), and from Caetano et al. (2022) for reptiles (GARD version 1.7). We obtained data on suitable habitat types for all species from the IUCN, which recognises 13 major terrestrial habitat types (iucnredlist.org/). We did not consider three habitat types: ‘introduced vegetation’, ‘other’, and ‘unknown’, and reclassified the remaining 10 habitat types into five classes—forest, non‐forest, agriculture, managed, and urban. These five classes match future land use projection categories (see also Powers and Jetz 2019 and Table S1). We considered all suitable habitat classes listed for each species. Overall, we obtained range maps and suitable habitat data for 29,657 tetrapod species (6407 amphibians, 9472 birds, 5161 mammals and 8617 reptiles).

Our analyses were conducted at a 24.125 × 24.125 km^2^ square grid in an equal‐area Behrmann projection. If a species' overall polygonal range was smaller than the area of a single grid cell (582.02 km^2^), we considered all the pixels overlapping with the species' range as part of its range. For species with larger ranges, we excluded cells with < 10% overlap with their original polygonal distribution from species distributions, following Murali et al. (2023).

We assessed land use changes over time (2015–2100) with the Land‐Use Harmonisation dataset (LUH2; available at https://luh.umd.edu/). LUH2 provides estimated land use changes divided into 12 categories at a 0.25 × 0.25° resolution, with each pixel containing proportions of several land use categories. We resampled these data to an equal‐area grid‐cell map of 24.125 × 24.125 km^2^ under Behrmann projection to match our species distribution data in an equal‐area projection grid. This dataset was previously used to study how future land use changes affect biodiversity (Asamoah et al. 2021; Cornford et al. 2023; Powers and Jetz 2019) and was found to have similar trends compared with a higher‐resolution dataset (Zeng et al. 2022). To match IUCN habitat categories and the LUH2 categories, we combined five types of crops in LUH2 to one class ‘agriculture’; primary and secondary forest into one class ‘forest’; primary and secondary non‐forest land were combined to one class of ‘non‐forest’; managed pasture and rangeland to ‘managed land’; and urban land as a class of its own (see Table S2). This reclassification enabled us to calculate habitat suitability per species per pixel between 2015 and 2100 (see below). Climate data for 1950–2100 were obtained from the NASA Earth Exchange Global Daily Downscaled Projections (NEX‐GDDP CMIP6) dataset (Thrasher et al. 2022) using the same resolution as the LUH2 dataset, following Murali et al. (2023).

### 2.2 Climate Suitability

Climate data were obtained from Murali et al. (2023), who quantified species' exposure to the frequency, duration and intensity of future extreme temperature events based on species‐range specific realised thermal limits. Murali et al. (2023) quantified species' realised thermal threshold as the spatial maximum of the top 99th percentile of daily maximum air temperature between 1950 and 2005 within each species range. Thermal events were defined as extreme when daily temperatures exceeded the species' thermal maximum for more than five consecutive days for each grid cell separately. Following Murali et al. (2023), we calculated the frequency, duration, and intensity of extreme temperature events for 1950–2005 and used each species' maximum of each metric to quantify species range exposure to climate extreme heat events in the future. Using these data, we classified grid cells (24.125 × 24.125 km) that will experience extreme events at a higher frequency, longer duration, or greater intensity than the maximum previously recorded as experiencing unsuitable climate conditions (see Murali et al. 2023, extended data Figure 1 for further details). In other words, grid cells within species range were designated as unsuitable when any one of the three metrics exceeded the species' historical maximum. We assessed climate conditions as suitable or unsuitable, considering exposure to extreme heat events per species per grid cell based on five General Circulation Models (GCMs) separately and on the median of these five models. To get the proportion of climatically suitable area within each pixel over time, we considered species' preferred habitat cover in 2015 (see below).

**FIGURE 1:**
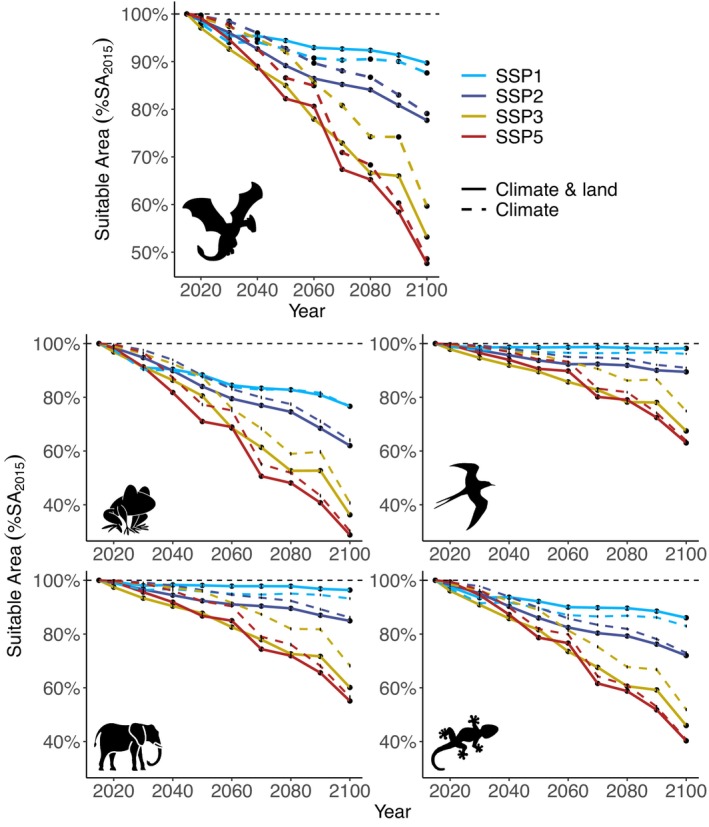
The average change in habitat suitability considering both extreme heat events and land use changes (solid line) and the expected change considering only extreme heat events (dashed line) for all taxonomic groups together (upper panel with dragon icon symbolising all tetrapod) and each taxa separately for each SSP‐RCP scenario (dragon icon made by Nsit and bird by Icongeek26 from www.flaticon.com; amphibian icon made by Luvdat; bird and reptile icons made by Freepik; mammal icon made by Icongeek26).

### 2.3 Suitable Area (SA) Calculations

For each species, we defined its ‘Suitable Area’: a combination of climatic suitability and suitable land use at each cell and time step. From each species' range, we calculated the area with suitable land use classes in 2015 and used that as our base comparison (initial Suitable Area SA_2015_ = 100%), assuming a suitable climate throughout all ranges in 2015. When evaluating changes in the percentage of Suitable Area for 2020–2100, we included suitable land uses and suitable climatic conditions (see above). If, at a certain point in time, a species had zero suitable area throughout its range (due to land use changes, extreme heat events, or both), we considered the species without any suitable area grid cells from that given year onwards, even if conditions became suitable later.

Some land conversions (e.g., natural vegetation recovering from previous human disturbances such as wood harvesting or agricultural abandonment) could potentially become suitable for some species. Even if the land will indeed become suitable, however, it may take considerable time before species can colonise it. We allowed for colonisation and recolonisation of any part of a species' current range as soon as the land use category changes to a suitable one for the species, regardless of connectivity or suitability in previous years. We acknowledge that this assumption may overestimate species' ability to persist, as dispersal limitations and connectivity constraints are known to strongly shape colonisation dynamics (Newbold et al. 2018; Thuiller et al. 2005). However, given the resolution of our analysis and the use of only five broad habitat categories from the LUH2 dataset, applying clade‐specific dispersal kernels or connectivity metrics would likely introduce a false sense of precision and be ecologically inconsistent with the underlying data. This may have led us to overestimate the availability of newly suitable areas and underestimate exposure to unsuitable conditions due to land use changes. However, it allows us to highlight areas of future potential suitability (gaining land; Figure 3 and Figures S5–S8) that might better fit future conservation plans. In this sense, our projections should be interpreted as a conservative ‘best‐case’ scenario that identifies opportunities for persistence rather than as realised predictions of species survival. Such potential benefits for biodiversity will depend, among other things, on land connectivity, management regimes, and the suitability of the surrounding area (Chazdon et al. 2009; Edwards et al. 2021).

The change in Suitable Area for each species (i), for each year (y), was calculated as the proportional change from its Suitable Area in 2015 (henceforth SA_2015_), following Equation (1) if species' Suitable Area increased in the future relative to SA_2015_ (due to land use changes) or Equation (2) if their future Suitable Area decreased relative to SA_2015_ (due to either land use or climate changes):

(1) $$\%\text{change in}\;{\mathrm{SA}}_{i,2015}\left[{\mathrm{SA}}_{i,y}>{\mathrm{SA}}_{i,2015}\right]=\left(1-\frac{{\mathrm{SA}}_{i,2015}}{{\mathrm{SA}}_{i,y}}\right)\times 100$$

(2) $$\%\text{change in}\;{\mathrm{SA}}_{i,2015}\left[{\mathrm{SA}}_{i,y}<{\mathrm{SA}}_{i,2015}\right]=\left(\frac{{\mathrm{SA}}_{i,y}}{{\mathrm{SA}}_{i,2015}}-1\right)\times 100$$

This represents the percentage of each species' range exposed to unsuitable conditions in the future compared to 2015, ranging between 0–200. These data are available at Vardi et al. (2025).

### 2.4 Statistical Analysis

For species whose future (i.e., year 2100) Suitable Area decreased relative to SA_2015_, we ran a zero–one inflated beta model to test whether species with initially smaller range sizes, or those with a higher IUCN threat category, are expected to experience more unsuitable conditions. We considered changes to Suitable Area per grid cell, in addition to per species. For this purpose, we calculated the average change in suitable area due to land use changes for all species in each cell. For climate suitability, as grid cells were either suitable or not, we considered the proportion of species that will be exposed to unsuitable climate conditions due to extreme heat events for each cell. With these two datasets, we created bi‐variate maps exploring the spatial overlap of land use changes and extreme heat events under future scenarios. All data analysis and plotting were conducted using R (R Core Team 2017).

## 3 Results

The combined effects of climate change and land use changes are projected to expose many species to unsuitable conditions through much or all of their current ranges by 2100, especially amphibians and reptiles (Figure 1 and Figure S1). In a sustainable, environmentally aware world (SSP1‐RCP2.6), exposure to unsuitable conditions due to extreme heat events will be lowest, with extreme heat events expected in 12.4% (±0.18 SE), on average, of species' initial Suitable Area in 2015 (SA_2015_) across all land vertebrates (23.3% ± 0.52, 3.8% ± 0.18, 6.8% ± 0.34, and 17% ± 0.39 for amphibians, birds, mammals, and reptiles respectively; Murali et al. 2023). Under this scenario, land use changes for anthropogenic activities are reduced, potentially increasing suitable areas and compensating for areas exposed to extreme heat events. Considering both factors, by 2100 the range exposed to unsuitable conditions could decrease by 2%–3%, on average, for all groups except for amphibians, which show similar range exposure compared to the effect of extreme heat events alone. Under SSP2‐RCP4.5 and SSP5‐RCP8.5 projections, land use changes have overall similar effects, with an average overall potential loss of Suitable Area due to land use changes in 2.1% and 2.3% of their SA_2015_, respectively, for all species by 2100. However, with high emission levels under SSP5‐RCP8.5, land vertebrates will experience, on average, unsuitable conditions due to the combined effects of extreme heat events and land use changes in 52.4% ± 0.22% of their 2015 ranges (compared to 22.3% ± 0.21% under SSP2‐RCP4.5). Differences between scenarios are mostly consistent across taxa (see Figure 1 and Table S4 for results per taxa).

Under SSP1‐RCP2.6, SSP2‐RCP4.5 and SSP5‐RCP8.5, exposure to extreme heat events will gradually increase and will be responsible for most changes in suitable areas. Under SSP3‐RCP7.0, land use changes may cause profound area unsuitability (preferred habitat type changing to unsuitable habitat) that compounds the impacts of extreme heat events far more than in other scenarios. Birds, mammals, and reptiles are expected to face decreases in their suitable area of 10% ± 0.2%, 13% ± 0.36%, and 11% ± 0.26% compared to their SA_2015_, 4–6 times higher than under SSP2‐RCP4.5 or SSP5‐RCP8.5 (1%–3%). Amphibians will face this exposure in almost 13% ± 0.3% of their SA_2015_, twice as much as under the SSP5‐RCP8.5 scenario (Table S1). Under all future scenarios, amphibians and reptiles are expected to be more exposed to unsuitable conditions across their ranges than birds and mammals (Figure 1). Even under the most optimistic scenario (SSP1‐RCP2.6), amphibians and reptiles are expected to face unsuitable conditions, on average, in over 23% ± 0.53% and 13% ± 0.43% of their 2015 ranges, respectively, due to the combined effects of extreme heat events and land use changes, compared to less than 2% and 4% for birds and mammals, respectively (Figure 1). These greater effects on amphibians and mammals may also be related to their relatively smaller range sizes (see Figures S2 and S3) as exposure to unsuitable conditions by 2100 was significantly higher for species with smaller initial range sizes, for amphibians and reptiles, and for species with higher IUCN threat categories (Table S3).

**FIGURE 2:**
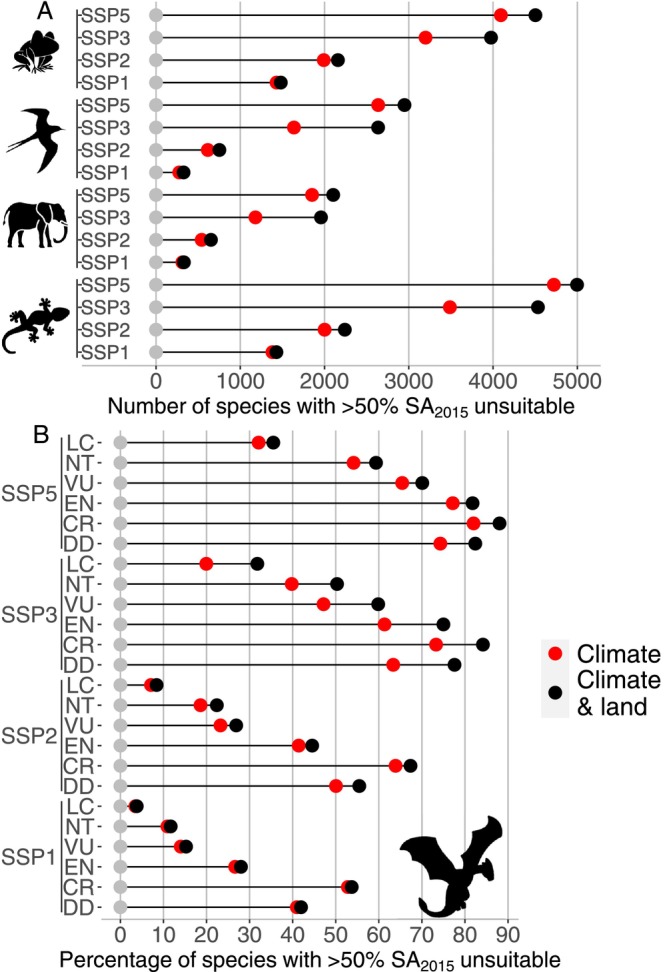
The number of species per taxa (A) and percentage of species in each IUCN threat category (B), that will be exposed to unsuitable conditions in at least half of their current (2015) suitable area due to climate extreme (red) or the combined effect of both land use and climate (black) under the four SSP‐RCP scenarios.

We found great differences in the number of species that are projected to face unsuitable conditions in most of their SA_2015_ by 2100 when comparing taxa and scenarios (Figure 2A). The combined effect of land use changes and extreme heat events will lead to 3333, 4242, 6607 and 7895 land vertebrate species facing unsuitable conditions throughout their entire current ranges (i.e., according to our analysis, they will face a high extinction risk) by 2100 under SSP1‐RCP2.6, SSP2‐RCP4.5, SSP3‐RCP7.0 and SSP5‐RCP8.5 respectively. We found that 3568, 5804, 13,103 and 14,552 species will be exposed to unsuitable conditions in at least half of their SA_2015_ by 2100 under these respective scenarios. Of these, 95.3%, 88.7%, 72.5% and 91.4% of species' suitable area losses are due to extreme heat events, and the remaining are due to land use changes or the combined effect of both factors. The proportion of such species increases with IUCN threat status. The percentages of species currently classified as Data Deficient and projected to experience unsuitable conditions across > 50% of their ranges resemble the percentages of Critically Endangered and Endangered species (Figure 2 and Figure S4) and exceed values for Vulnerable species. On average, the percentage of area expected to be exposed to unsuitable conditions is greater for CR, EN, and DD species and lowest for LC species (Figures S3 and S4). Throughout all scenarios, we see a more‐than‐additive effect of extreme heat events and land use changes, with more species expected to be affected across large areas of their current ranges (Figure S4).

**FIGURE 3:**
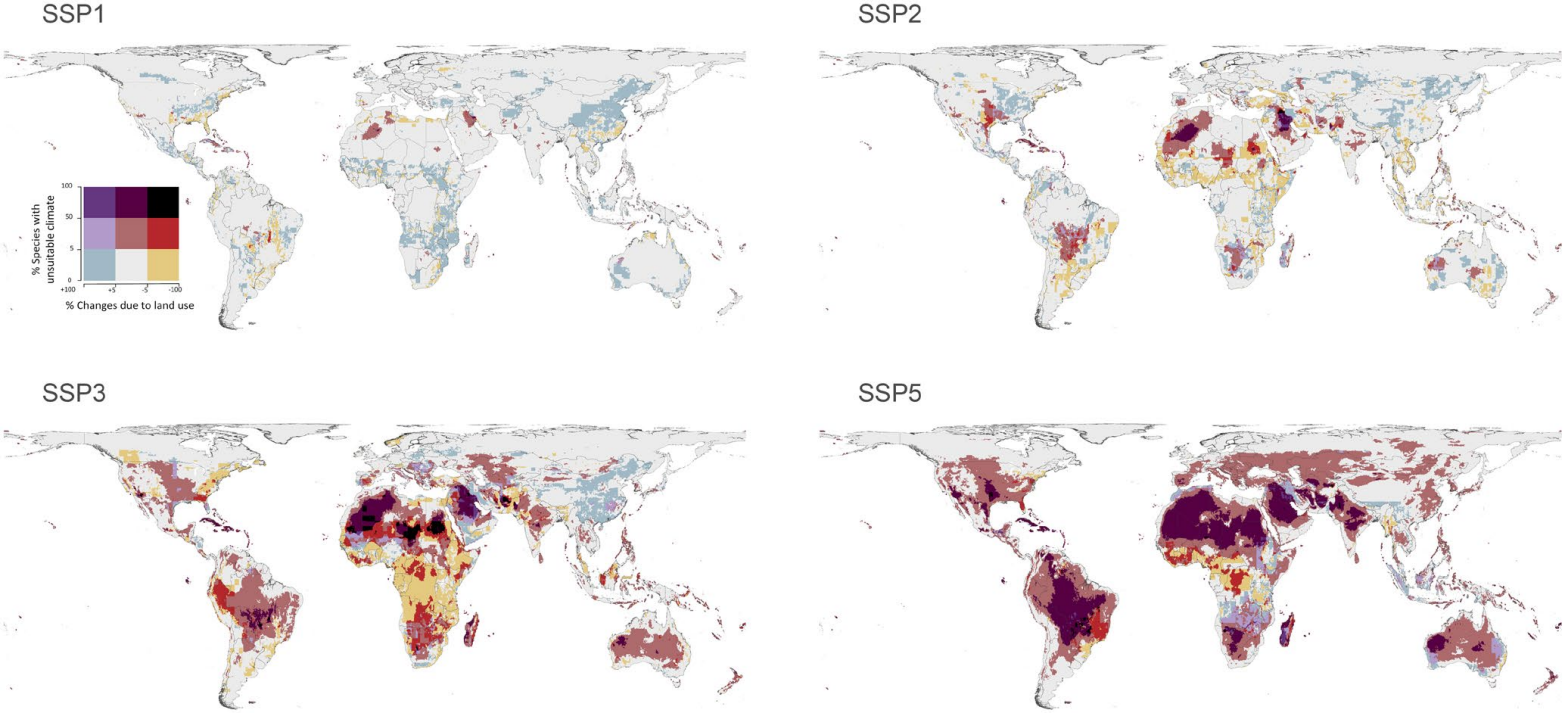
Bi‐variate map exhibiting areas exposed to unsuitable conditions based on mean values of 2091–2100 for each of the four SSP‐RCP scenarios (compared to 2015). The *y*‐axis indicates the proportion of species within each grid cell (24.125 × 24.125 km^2^) that are expected to be exposed to climate extremes. The *x*‐axis depicts the averaged proportional change in land use suitability in each grid cell in comparison to 2015; positive values indicate an overall potential expansion of suitable areas, whereas negative values indicate the loss of suitable habitat due to land use changes. See Figures S4–S7 for maps of each taxonomic group separately. Map lines delineate study areas and do not necessarily depict accepted national boundaries.

We found potential gains of suitable area due to land use changes across all continents under SSP1‐RCP2.6 (Figure 3). In this scenario, exposure to unsuitable climate is geographically restricted (mainly to Algeria and Iraq), with scattered unsuitable areas due to land use changes and little overlap between land use changes (either positive or negative) and extreme heat events. Under SSP2‐RCP4.5, extreme heat events are far more widespread. In this scenario, more than 50% of species (per grid cell) will face extreme heat events in large areas of North Africa and the Middle East. Land use changes may still benefit species in multiple areas across all continents, but the potential loss of suitable land is far more extensive than under SSP1‐RCP2.6, especially in Africa (Sudan, South Sudan, Ethiopia, Somalia, Kenya, Nigeria, Benin, Mali, Senegal), the Middle East (Turkey, Iraq and Iran), and South America (Brazil, Paraguay, and Argentina). Under SSP3‐RCP7.0 and SSP5‐RCP8.5, exposure to unsuitable conditions is expected to be much more severe and occur across all continents. Under SSP3‐RCP7.0, unsuitable land and unsuitable climate will overlap across North Africa, Afghanistan and Peru. Some potential gains due to land use changes are expected in China, the Middle East, and Eastern Europe, but many species across Africa are predicted to experience unsuitable conditions due to either extreme heat events or land use changes. Under SSP5‐RCP8.5, most species will face unsuitable climate conditions in vast areas across Brazil, Bolivia and Paraguay, North Africa, the Middle East, India and Western Australia. Species are expected to experience extreme heat events in the future even in potentially new suitable areas due to land use changes, mostly in Africa and Australia. The spatial patterns are relatively similar across vertebrate taxa (Figure 3 and Figures S5–S8). Considering the absolute number and proportion of species expected to be negatively affected by 2100 by both extreme heat events and land use changes, subtropical areas are highlighted as most threatened (Figures S9 and S10). Many species in the Caribbean Islands and a few areas across South America are already expected to be exposed to unsuitable conditions under SSP1 and SSP2.

## 4 Discussion

Overall, our analyses suggest that thousands of land vertebrate species will face extreme heat events and/or unsuitable habitats throughout most, or even all, of their current ranges in the coming decades, emphasising the critical importance of considering multiple interacting threats in conservation planning (Rillig 2025). We found major differences in effects between projected future scenarios (Figures 1, 2, 3). Higher RCP leads to greater exposure to extreme heat events, and high social inequality and conflicts (SSP3) lead to more negative effects of land use changes. Of the scenarios examined here, SSP1‐RCP2.6 is the only one upholding obligations to the Paris Agreement to limit warming to below 2°C (United Nations 2015). Following concerns regarding the ability to reach this commitment (unfccc 2022), global efforts must ensure actions to strengthen resilience and reduce vulnerability to climate change. Without active intervention, many species are likely to experience severe population declines or even go extinct. This is particularly true for species already considered threatened (VU, EN, CR) by the IUCN, species defined as Data Deficient (Figure 2), and amphibians and reptiles, which are already the most threatened groups of vertebrates (Cordier et al. 2021; Figures 1 and 2, Figure S3). Ectotherms are known to be particularly susceptible to environmental changes (Hayden Bofill and Blom 2024), but this could also be due to their smaller ranges to begin with (Figures S2 and S3, Table S3; Newbold et al. 2018; Thuiller et al. 2005).

Our findings align with recent assessments showing that the combined effects of climate and land use change are predicted to lead to substantial cumulative losses for vertebrate species (Prieto‐Torres et al. 2021; Williams et al. 2022). Considering extreme heat events, Murali et al. (2023) showed that mid‐latitudinal areas will be more exposed to future extreme heat events than tropical regions. They found that most species in these areas will experience extremes in most parts of their range, with little heterogeneity to help buffer their impact (see also Pinsky et al. 2019). Furthermore, Anderson et al. (2022) showed that the warming tolerance of desert lizards was narrower than that of their tropical kin, further suggesting mid‐latitudinal areas are at greater risk from the effects of climate change. Adding the effect of land use changes, which is weaker in comparison to extreme heat events, our results align with Murali et al. (2023), highlighting subtropical areas in North Africa, the Middle East, ans parts of South America as those with the greatest negative impact, considering the number and proportion of species affected (Figures S9 and S10).

Our results demonstrate the potential harmful effects land use changes can continue to impose on biodiversity. These detrimental changes are particularly noticeable in the Global South, as cropland areas increase at the expense of natural habitats (Winkler et al. 2021). Species can be negatively affected by extreme heat events in one part of their range and by land use changes in another, leading to a more‐than‐additive combined effect (Figure 3). For example, under SSP3‐RCP7.0, the African bush viper, *Atheris broadleyi*, is expected to lose 81% of its 2015 suitable area due to land use changes and 76% due to extreme heat events by 2100. Given the incomplete spatial overlap between the two factors, when these two drivers are combined, the species is expected to face unsuitable conditions in 98% of its range (see Figure S11). Such trends of exposure to extreme heat events and land use changes in neighbouring areas are very noticeable under SSP3‐RCP7.0 in Africa, the Middle East and South America (Figure 3). In parts of these regions, climate and land use changes spatially overlap, leaving many species experiencing a double threat.

Extreme heat events and land use changes can interact in multiple ways and create feedback loops that may further adversely affect populations (Murali et al. 2023; Prevedello et al. 2019; Williams et al. 2022). Habitat loss and fragmentation can lead to smaller, isolated populations that are more susceptible to climate change or other anthropogenic stressors (Chan et al. 2024; Guo et al. 2018; Mantyka‐pringle et al. 2012). Similarly, climate change, particularly extreme heat events, can further exacerbate the risks of land use changes by accelerating habitat degradation, increasing thermal stress and limiting refugia availability (Lauck et al. 2023; Murali et al. 2023; Segan et al. 2016). These interactions limit species' ability to disperse and migrate, forcing them into suboptimal conditions (Chen et al. 2022; Sales et al. 2019). Such feedback loops can amplify biodiversity loss by diminishing genetic and functional diversity, and ecosystem resilience, ultimately weakening the capacity of species to recover from or adapt to rapid environmental changes (Brando et al. 2025; Faillace et al. 2021). For example, Bolochio et al. (2020) mapped global patterns of amphibian functional diversity and identified two ectomorph hotspots in Brazil crucial for conservation: the central Amazon and the southern Atlantic Forest. Under our projections, the Atlantic Forest is expected to face both extreme heat events and land use changes under most future scenarios, emphasising the potential loss of phylogenetic and functional diversity that could undermine ecosystem stability, productivity, and resilience (González‐Maya et al. 2025).

Additional stressors we did not consider in our analyses, such as interspecific interactions, other climate extreme events (e.g., wildfires, droughts), and anthropogenic pressures (e.g., novel pathogens and diseases, pollution, invasive species), can interact with extreme heat events and land use changes in complex ways that further exacerbate biodiversity loss. For example, habitat fragmentation may lead to increased competition among species, heightening stress during extreme heat events (Faillace et al. 2021). Invasive species, which could be facilitated by climatic extremes (Diez et al. 2012), can further disrupt native species interactions and accelerate biodiversity declines (Bhat et al. 2024; Vetter et al. 2020). Introduced pathogens and diseases, along with chemical and light pollution, further degrade ecosystems by increasing vulnerability to extreme climatic events and land‐use pressures (Carlson et al. 2025; Pfenning‐Butterworth et al. 2024). For example, land‐use changes can increase the risk of emerging zoonotic diseases by altering the dynamics of pathogen transmission between humans and wildlife (Roque et al. 2023). When considered all together, these environmental changes can trigger losses in phylogenetic and functional diversity and precipitate secondary extinctions (Ceron et al. 2023; Mahecha et al. 2024). Thus, our projections potentially represent conservative estimates of future risks, since compounding threats are likely to further increase extinction risk and reduce the effectiveness of conservation interventions.

Species responses to these stressors can be mediated by a complex interplay of microhabitats, behavioural flexibility, and physiological adaptations that can collectively enhance resilience. The quality and availability of microhabitats can play a substantial role in assisting species during extreme heat events, especially in anthropogenically modified land, as studies show microhabitats can reduce local temperatures by up to 5°C (Scheffers et al. 2014). For example, patches of shrubs and trees across a semi‐natural area provided crucial microclimate refugia for little bustards ( *Tetrax tetrax* ) during extreme heat conditions (Ramos et al. 2023). Behavioural flexibility, such as shifts in microhabitat use or activity timing, can play a crucial role in adapting to land use changes as well as changing climate (Ameca et al. 2023; Beever et al. 2017; Vardi and Berger‐Tal 2022). For example, the American pika ( *Ochotona princeps* ), alters its foraging behaviour, habitat use, and activity periods to exploit cooler microhabitats and avoid heat stress in a warming climate (Beever et al. 2017). Physiological flexibility can complement such behavioural strategies by allowing adjustments in metabolism, water loss or hormone secretion to cope with environmental changes (Seebacher et al. 2023; Taff et al. 2024; Vardi et al. 2023).

Protected Areas (PAs) can also mitigate biodiversity loss, increasing their importance in a changing world (Mi et al. 2023). However, some studies question their effectiveness. Amphibians, for example, are poorly represented within PAs (Nori et al. 2015), and general assessments suggest overestimation of how well PAs represent threatened vertebrates (Cordier et al. 2025). Our results show that extreme heat events can affect biodiversity also inside PAs. Under SSP5‐RCP8.5 for example, many PAs globally could be affected by extreme heat events at higher frequencies, duration, or intensity than in the past (Figure 3). Even under SSP2‐RCP4.5, PAs in Australia, Brazil, Bolivia and Paraguay could potentially suffer from such extremes. Many PAs surrounding areas will face climate and land use changes, which can negatively affect biodiversity within PAs as well (Auliz‐Ortiz et al. 2024). This, according to our results, is particularly true in Madagascar, South America, the East United States and the Caribbean, where extensive land use changes are expected under all future scenarios. These biodiversity‐rich areas where many species are expected to be exposed to unsuitable conditions, are particularly vulnerable under SSP3‐RCP7.0 (Figure S9). Extreme heat events are also predicted to impact newly identified hotspots for threatened vertebrates in Madagascar, the Caribbean and Central and South America (Huais et al. 2025), underscoring a need for conservation prioritisation. Considering predicted climate and land use pressures, strengthening and expanding well‐connected protected areas, alongside strategic conservation priorities that focus on ecological representativeness and adaptive management, will be vital to buffer against future biodiversity loss and sustain resilient ecosystems worldwide (Cordier et al. 2025; Prieto‐Torres et al. 2022). This aligns with broader conservation‐planning results that identified gaps in effectiveness and priority‐setting under global change (Strassburg et al. 2017; Triviño et al. 2018; Vergara‐Tabares et al. 2020).

We predict that extreme heat events will become the leading force affecting area suitability for species by 2100, with land use changes having weaker impacts (Table S4). However, we may have underestimated the effects of land use changes due to several reasons. First, the above‐mentioned expansion throughout a species' range, regardless of connectivity, previous suitability, or dispersal abilities, may have led to optimistic assessments of land use changes. Limiting species dispersal in time (waiting a few years after a grid cell becomes suitable before being able to colonise it) or space would probably lead to more alarming projections, particularly for species in highly fragmented landscapes and species with limited dispersal abilities (Inman et al. 2023; Newbold 2018). While we recognise these as major sources of uncertainty, with limited knowledge regarding dispersal abilities and connectivity (Lancaster et al. 2024), incorporating any general assumptions would potentially introduce more errors and uncertainties. Second, suitability based on land use changes considered proportional values of suitable habitat, whereas climate suitability was assessed as either suitable or unsuitable, increasing the apparent effect of climate compared to land use changes.

Third, land use scenarios often make optimistic assumptions about agricultural yields and technological improvements (Newbold 2018), ignoring the effect of climate change on agricultural yield, which may further lead to overoptimistic estimations. Even under the given projections, future land use projections do not reflect changes in land use intensity and land management regimes, which can greatly influence biodiversity and habitat suitability (Millard et al. 2021; Titeux et al. 2016). All these assumptions limit the estimated effects we identified. Land use intensity is estimated to be responsible for a quarter of the negative effects of land use changes on biodiversity and can further impact biodiversity even without additional land conversion (Semenchuk et al. 2022). Thus, more realistic trajectories of agricultural expansion and land use intensity could substantially increase the impacts of land use we found. Finally, our classification of suitable habitats into five broad classes, derived from the LUH2 dataset (see Section 2), may be too coarse. Finer habitat distinctions would potentially alter the results, putting greater emphasis on the detrimental effects of land use changes. Crucially, it probably leads to overestimating species' suitable areas (Kuemmerle 2024). For example, we considered ‘rural gardens’ (IUCN category 14.4) under ‘urban area’. Thus, we estimated the range of species inhabiting rural gardens but not built urban areas, as the combined range of gardens and built areas. Still, our results resemble those of previous studies in showing the most extensive land use changes under SSP3 and predicting, on average, a > 10% decrease in suitable area under this scenario (Powers and Jetz 2019; Simkin et al. 2022).

We highlight the importance of considering both land use and biologically meaningful aspects of climate change to better predict future environmental changes' effects on species distribution globally. Our results can help guide future conservation policy efforts, highlighting the immense potential losses to biodiversity if no mitigation actions take place. With climate change expected to become the main threat to biodiversity (Strona and Bradshaw 2022; Urban 2015), considering expected land use changes on top of climate change can further inform conservation science and efforts as to the species and areas most in need of conservation. It can further highlight areas where such efforts can be most fruitful. These unprecedented challenges require immediate and global action planning to ensure the safeguarding of biodiversity as well as human well‐being.

## Author Contributions

**Reut Vardi:** conceptualization, data curation, formal analysis, visualization, writing – original draft, writing – review and editing. **Gopal Murali:** conceptualization, data curation, formal analysis, writing – review and editing. **Gabriel Henrique de Oliveira Caetano:** writing – review and editing. **Uri Roll:** conceptualization, funding acquisition, writing – review and editing. **Shai Meiri:** conceptualization, funding acquisition, writing – review and editing.

## Funding

This work was supported by the Israel Science Foundation (ISF; grant no. 611/23).

## Conflicts of Interest

The authors declare no conflicts of interest.

## Supporting information


**Figure S1:** gcb70625‐sup‐0001‐Supinfo.docx.

## Data Availability

The data that support the findings of this study are available on FigShare at https://figshare.com/s/f08e170a2b032a329b6f.
