# Supplementary material for: Effects of Future Climate Extreme Heat Events and Land Use Changes on Land Vertebrates
Source: Glob Chang Biol. 2025 Dec 9;31(12):e70625. doi: 10.1111/gcb.70625 (PMC12687109; doi:10.1111/gcb.70625)
Supplement: Supplementary file 1 — Figure S1: gcb70625‐sup‐0001‐Supinfo.docx. [file GCB-31-e70625-s001.docx]

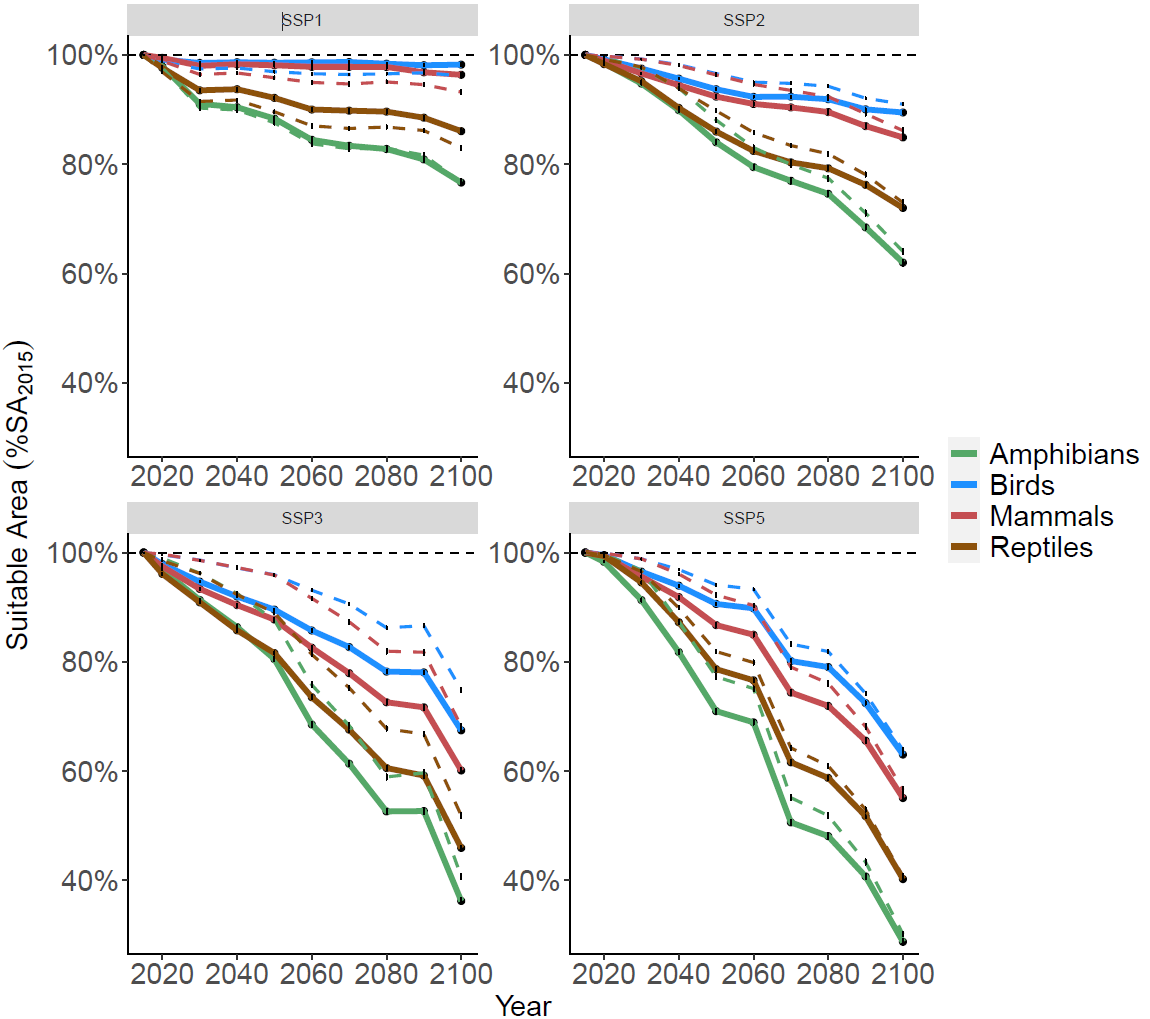


Fig. S1: The average change in habitat suitability considering both climate extremes and land use changes (solid line) and the expected change considering only climate extreme (dashed line) for each SSP-RCP scenario and each taxa (amphibians, birds, mammals, and reptiles).

Fig. S2: The proportion of area expected to remain suitable in 2100 for A) each taxonomic group (amphibians, birds, mammals, and reptiles); B) IUCN threat category; and C) quartiles of initial 2015 suitable area size, with 1 representing the species with the smallest range sizes (up to 4225 km2), and 4 the ones with the biggest (430000-49608210 km2). Boxplots display the medians and interquartile range. Species expected to gain suitable area due to land use changes and increase their overall 2015 suitable area were excluded from the analysis (a zero-one inflated beta model, see Table S4). Letters on top of box plot represent significant differences between groups based on post-hoc Tukey comparison


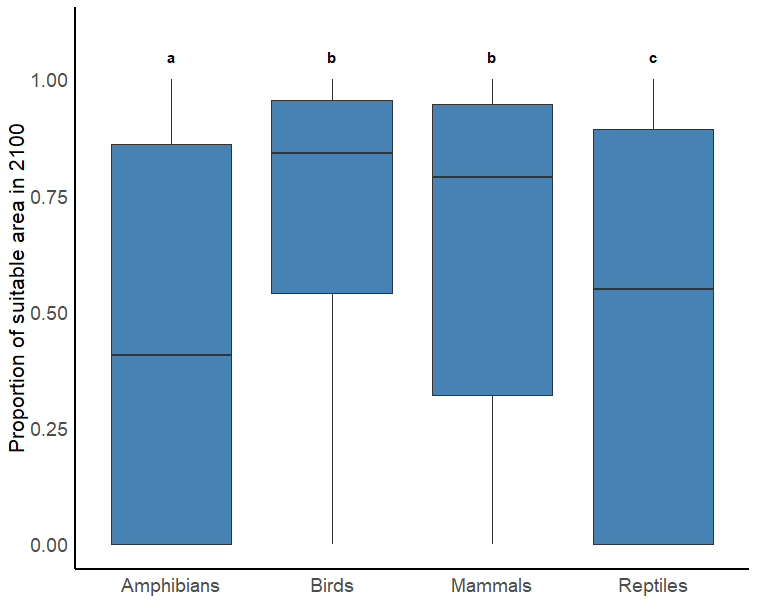

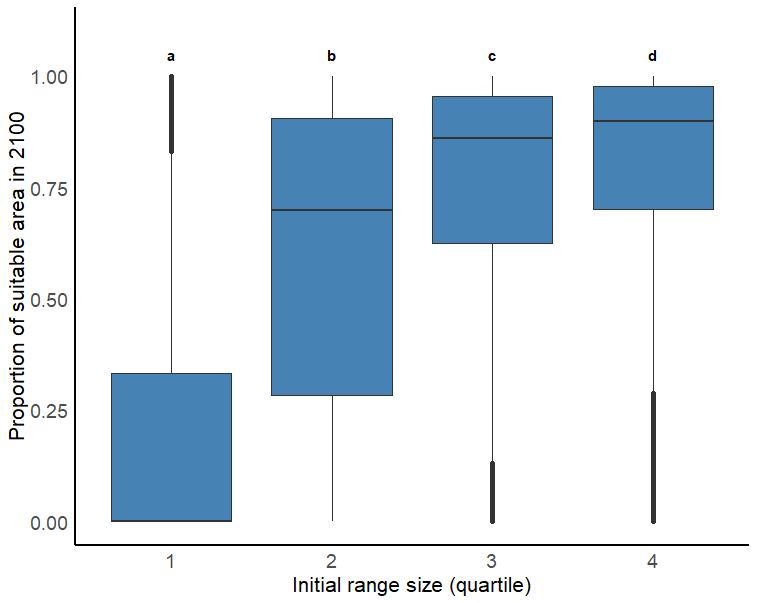

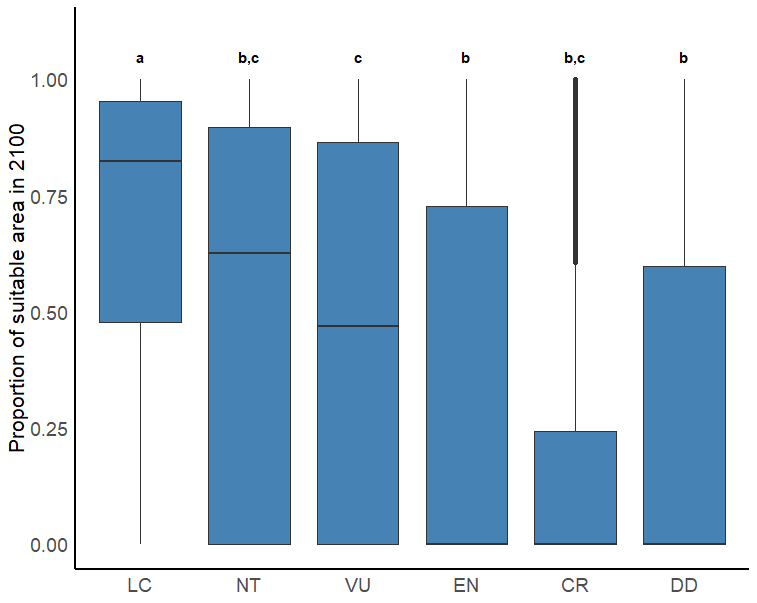


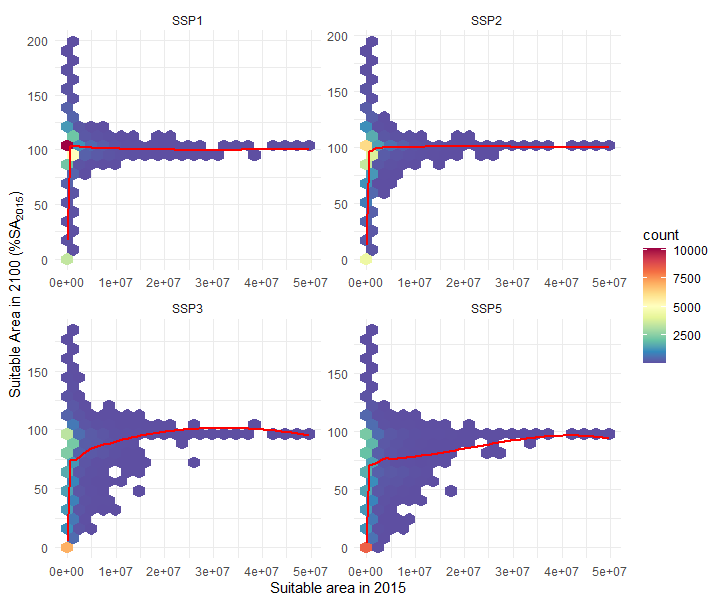


Fig. S3: A hexbin plot showing the distribution of data points for species change in Suitable Area in 2100 as a function of their suitable area in 2015 per SSP-RCP scenario.


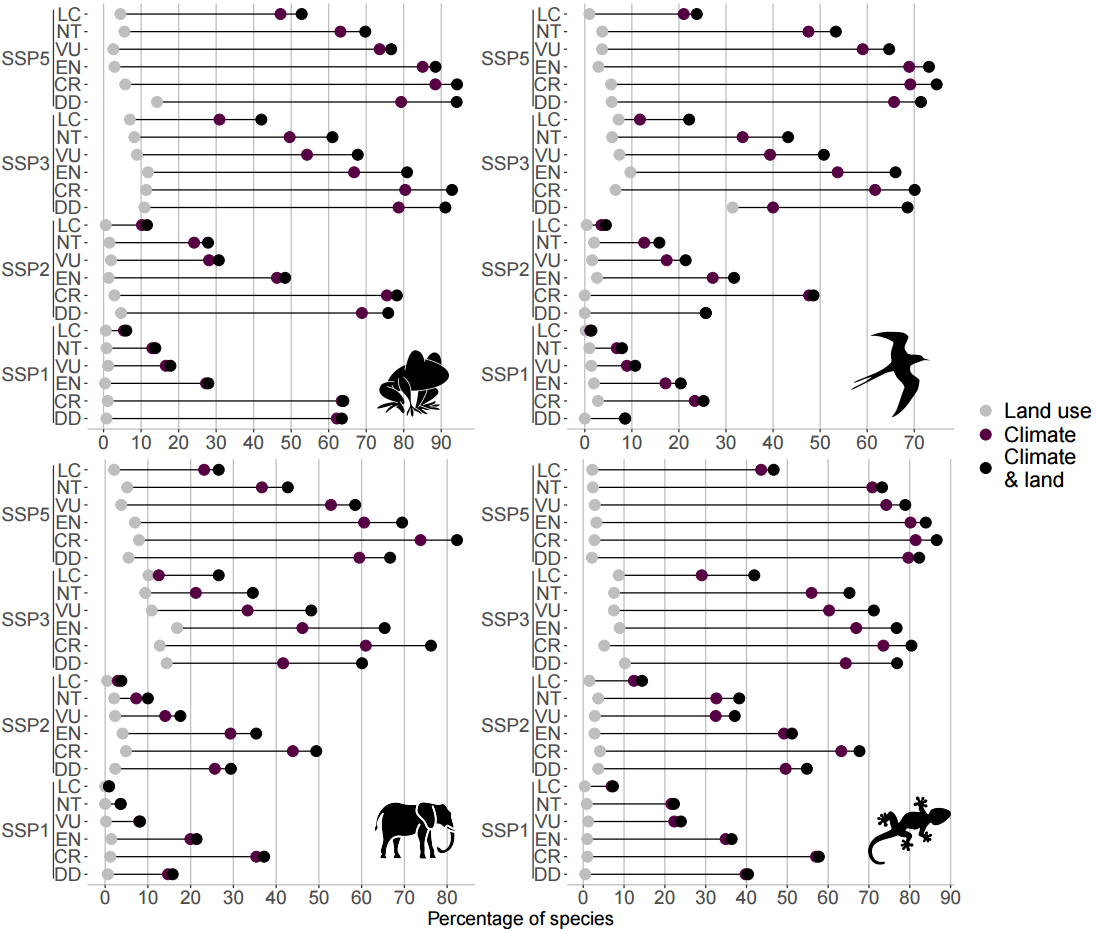

Fig. S4: The percentage of species in each IUCN threat category that will be exposed to unsuitable conditions in at least half of their current (2015) suitable area due to land use changes (grey), climate extreme (purple), or the combined effect of both land use and climate (black) under the four SSP-RCP scenarios for each taxonomic group.


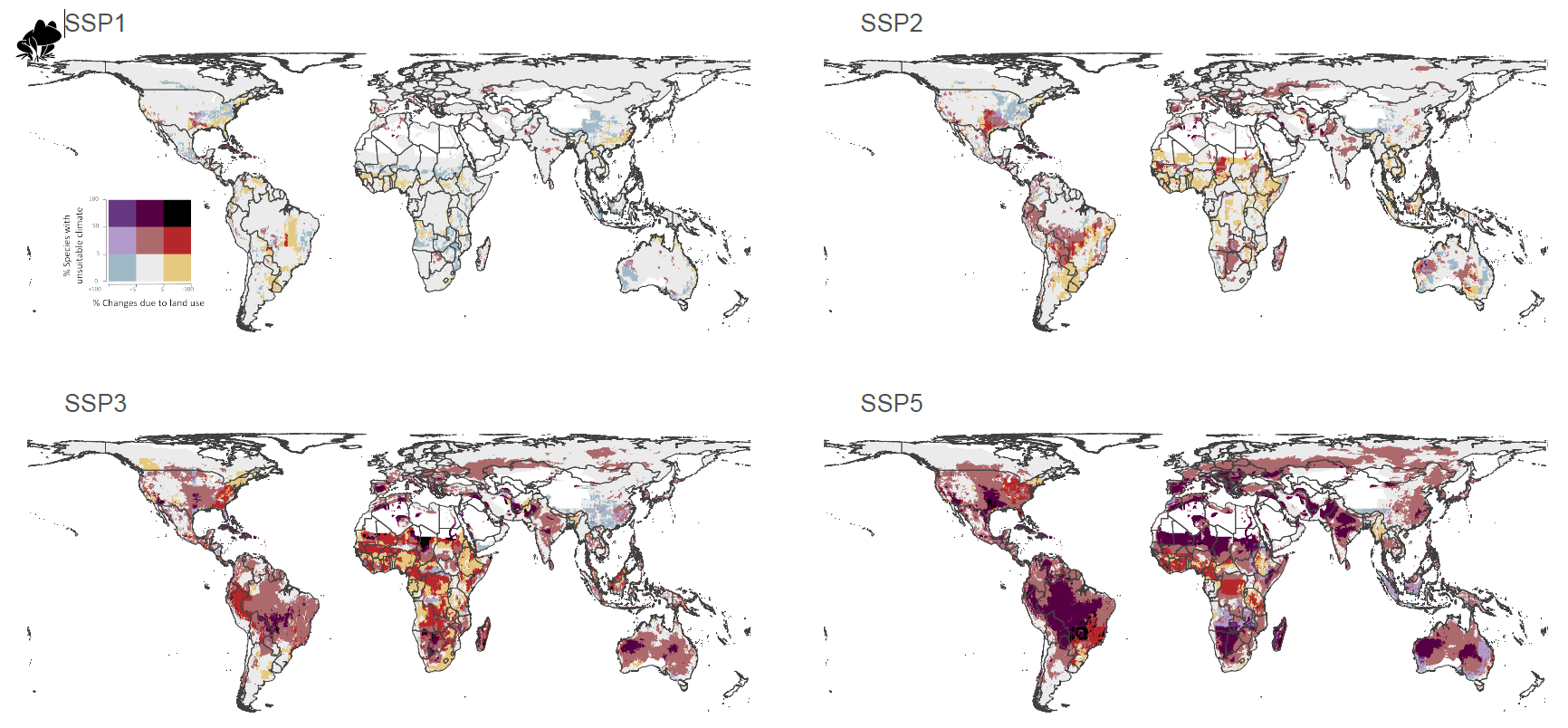
Fig. S5: Bi-variate map exhibiting areas exposed to unsuitable conditions based on mean values of 2091-2100 for each of the four SSP-RCP scenarios for all amphibians (6,407 species). The Y-axis indicates the proportion of species within each grid cell (24.125 x 24.125 km^2^) that are expected to be exposed to climate extremes. The x-axis depicts the averaged proportional change in land use suitability in each grid cell in comparison to 2015; positive values indicate an overall potential expansion of suitable areas, whereas negative values indicate the loss of suitable habitat due to land use changes.


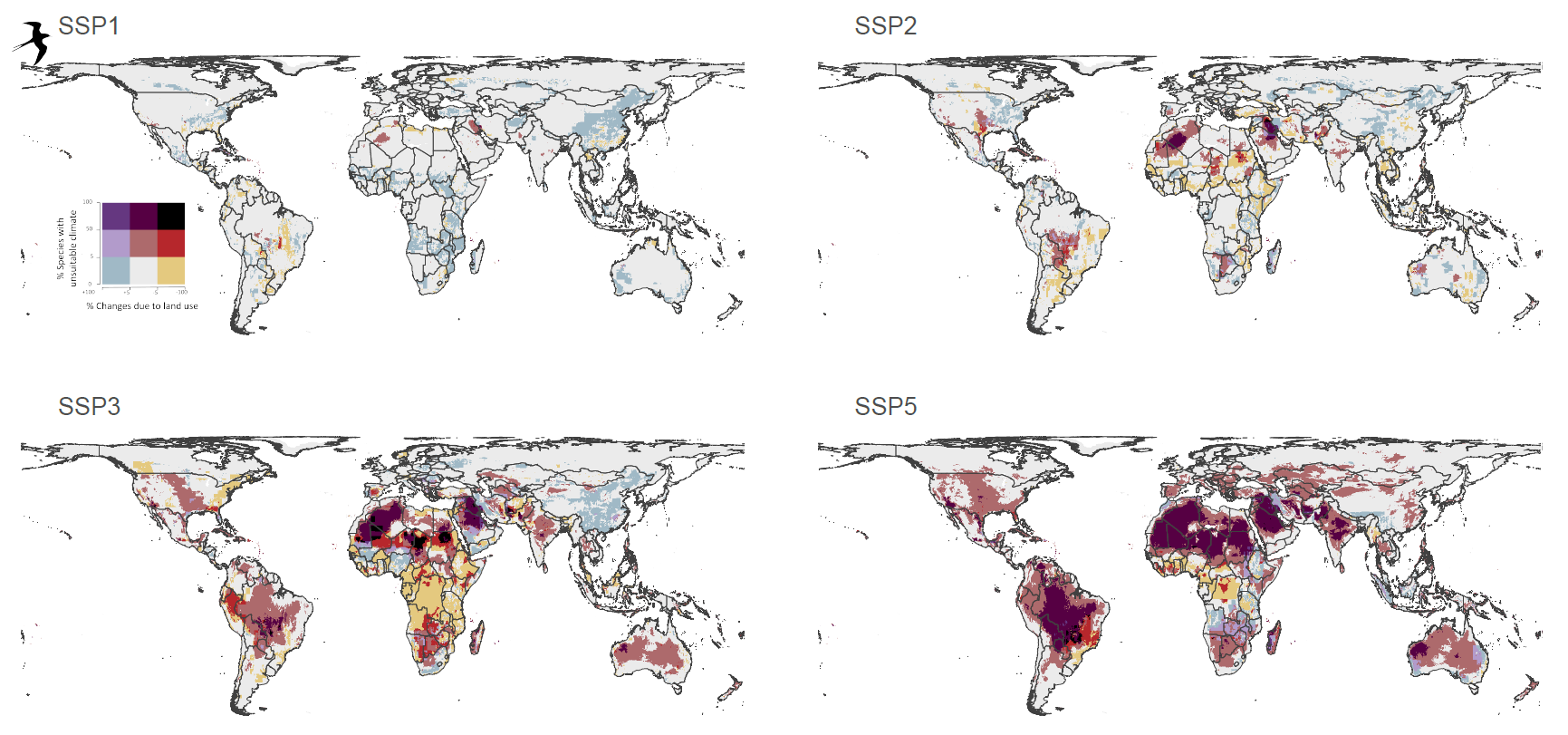
Fig. S6: Bi-variate map exhibiting areas exposed to unsuitable conditions based on mean values of 2091-2100 for each of the four SSP-RCP scenarios for all birds (9,472 species). The Y-axis indicates the proportion of species within each grid cell (24.125 x 24.125 km^2^) that are expected to be exposed to climate extremes. The x-axis depicts the averaged proportional change in land use suitability in each grid cell in comparison to 2015; positive values indicate an overall potential expansion of suitable areas, whereas negative values indicate the loss of suitable habitat due to land use changes.


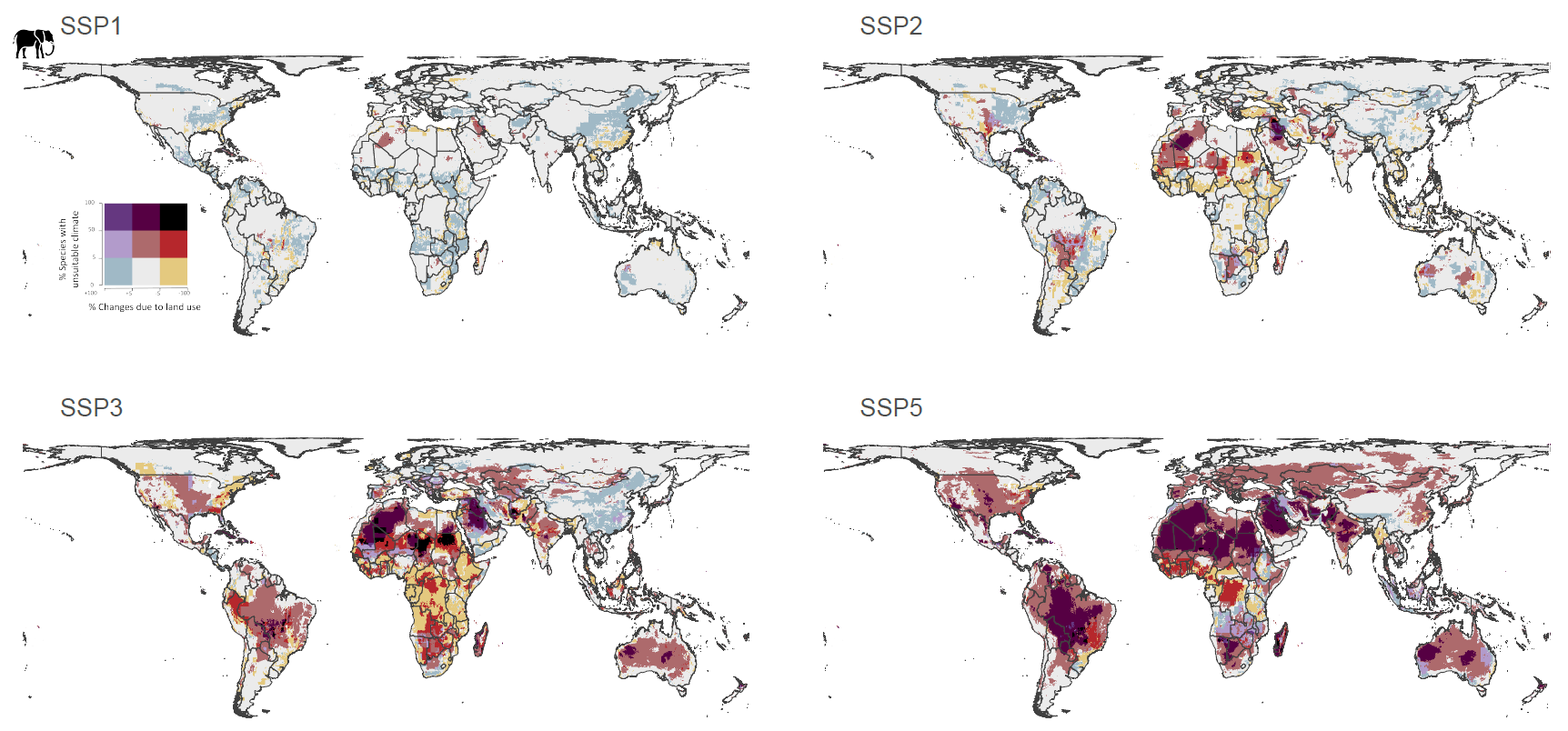
Fig. S7: Bi-variate map exhibiting areas exposed to unsuitable conditions based on mean values of 2091-2100 for each of the four SSP-RCP scenarios for all mammals (5,161 species). The Y-axis indicates the proportion of species within each grid cell (24.125 x 24.125 km^2^) that are expected to be exposed to climate extremes. The x-axis depicts the averaged proportional change in land use suitability in each grid cell in comparison to 2015; positive values indicate an overall potential expansion of suitable areas, whereas negative values indicate the loss of suitable habitat due to land use changes.


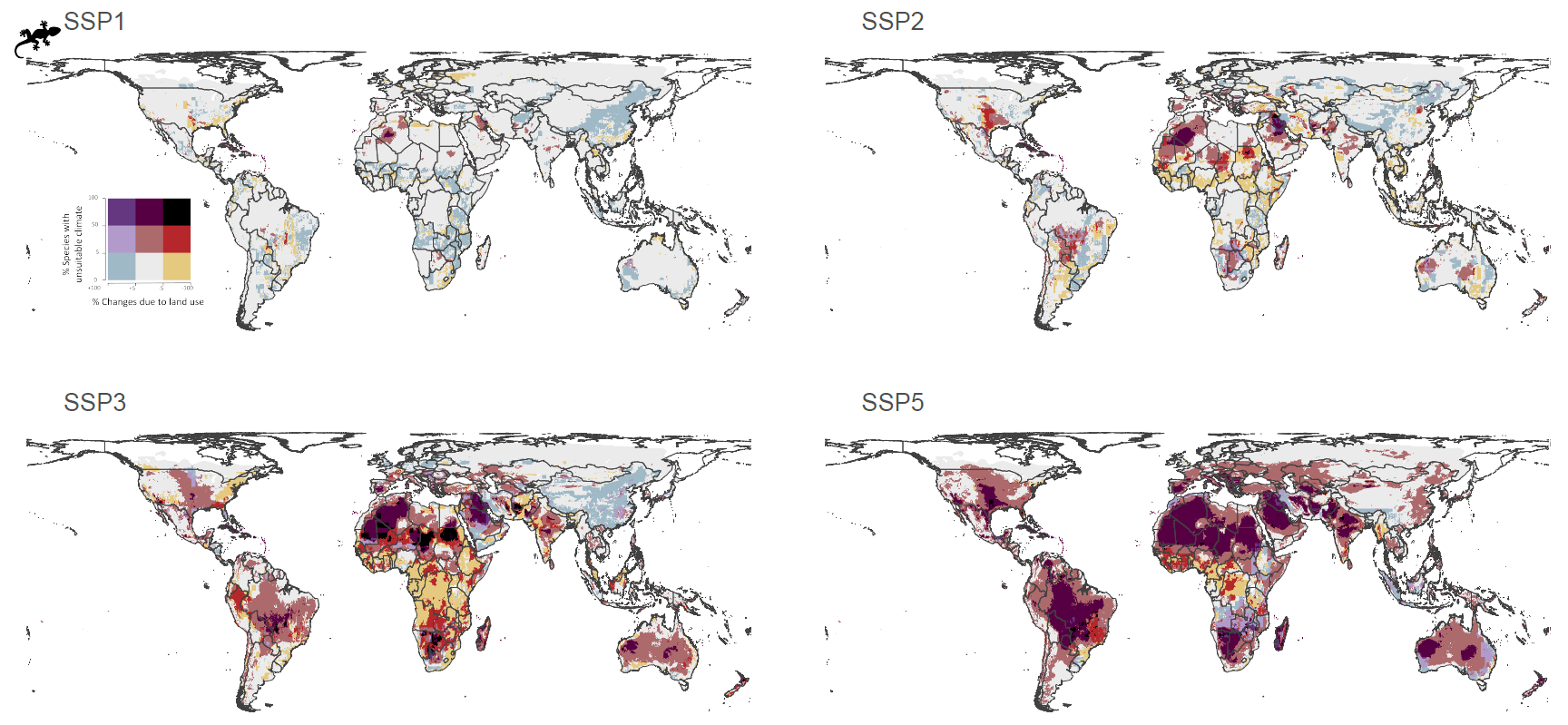
Fig. S8: Bi-variate map exhibiting areas exposed to unsuitable conditions based on mean values of 2091-2100 for each of the four SSP-RCP scenarios for all reptiles (8,617 species). The Y-axis indicates the proportion of species within each grid cell (24.125 x 24.125 km^2^) that are expected to be exposed to climate extremes. The x-axis depicts the averaged proportional change in land use suitability in each grid cell in comparison to 2015; positive values indicate an overall potential expansion of suitable areas, whereas negative values indicate the loss of suitable habitat due to land use change

Fig. S9: The number of species per grid cell expected to be exposed in at least 50% of their suitable area and the corresponding latitudinal patterns for the mean species exposed per pixel for each scenario.


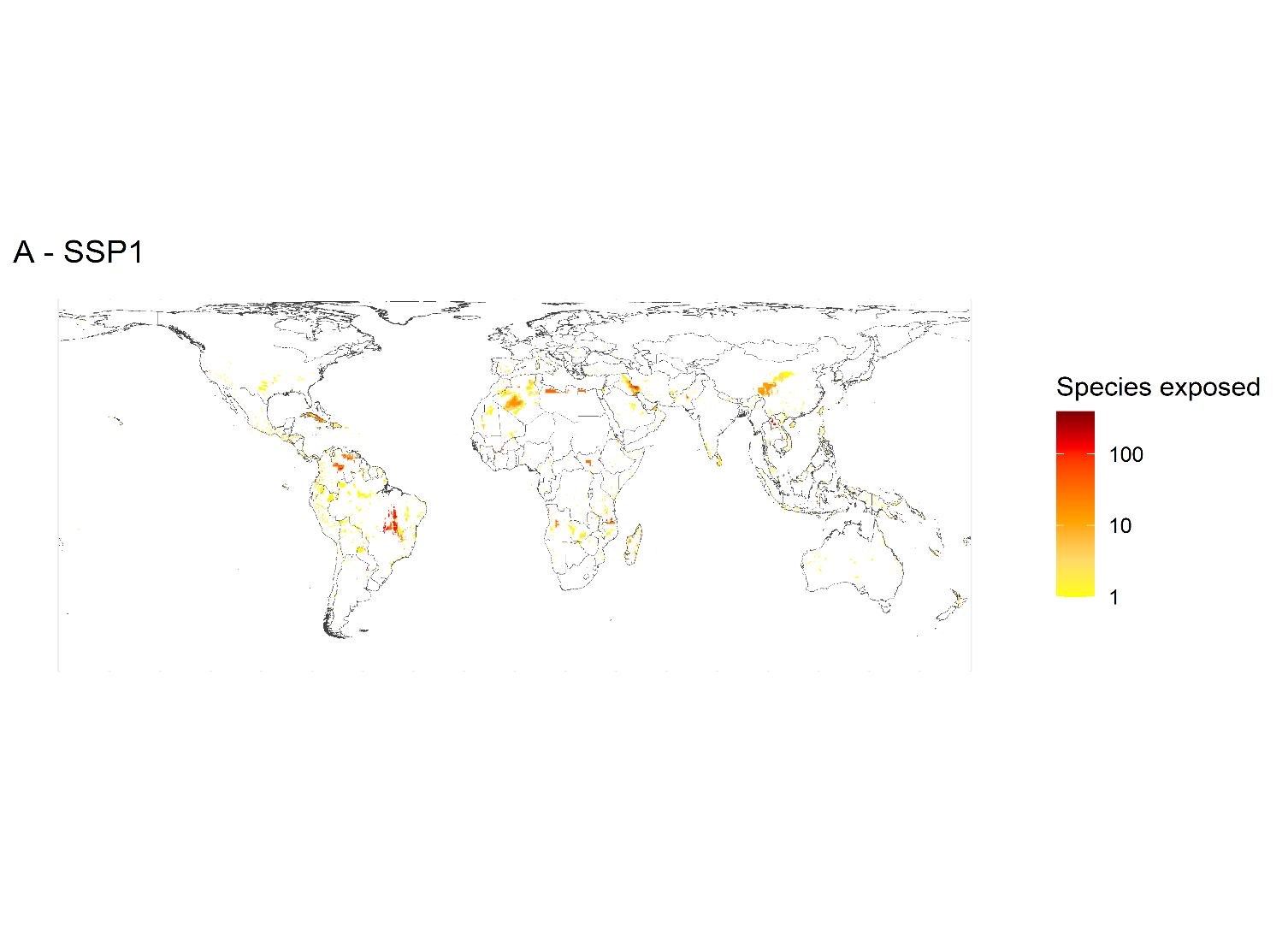

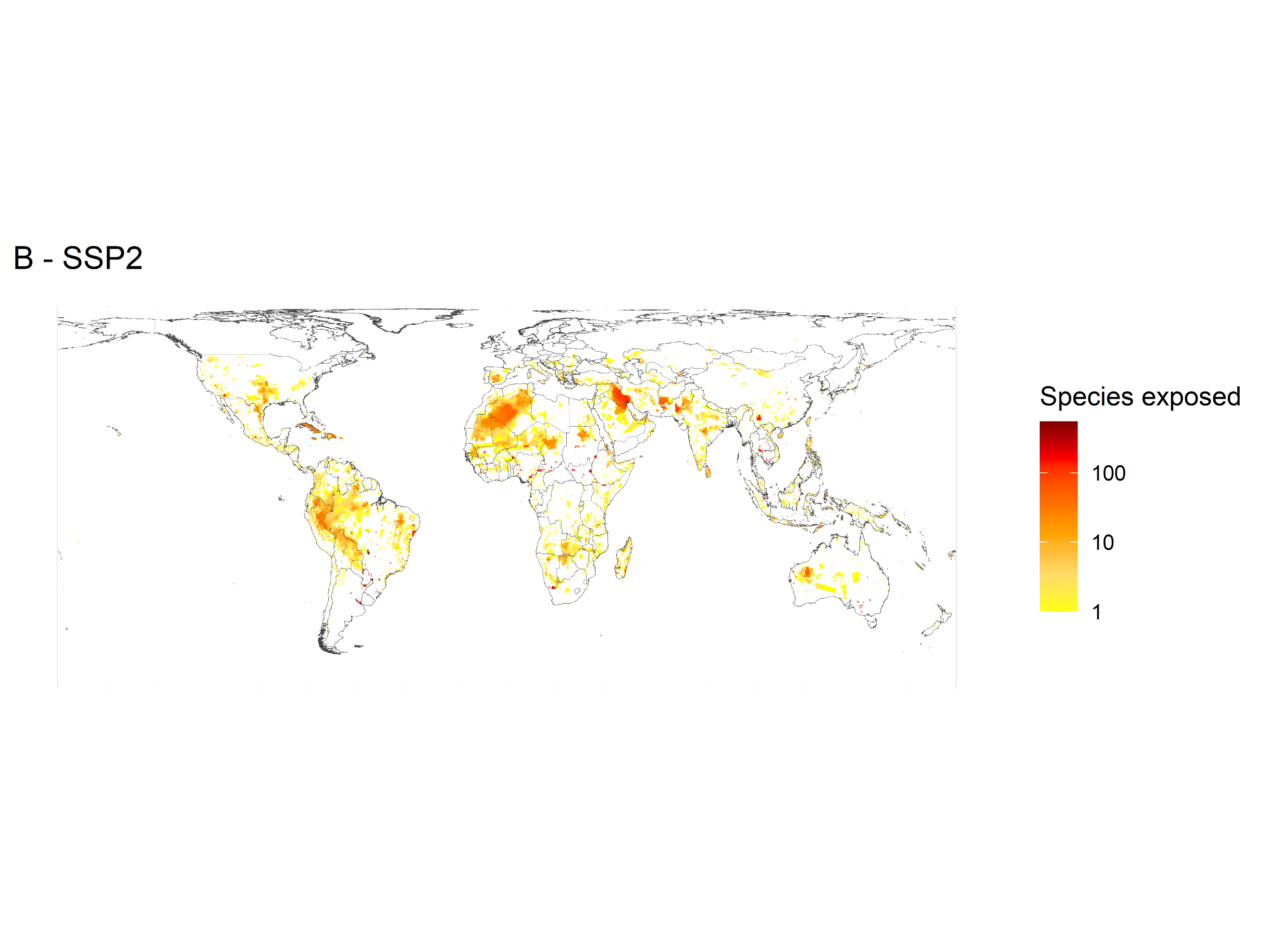

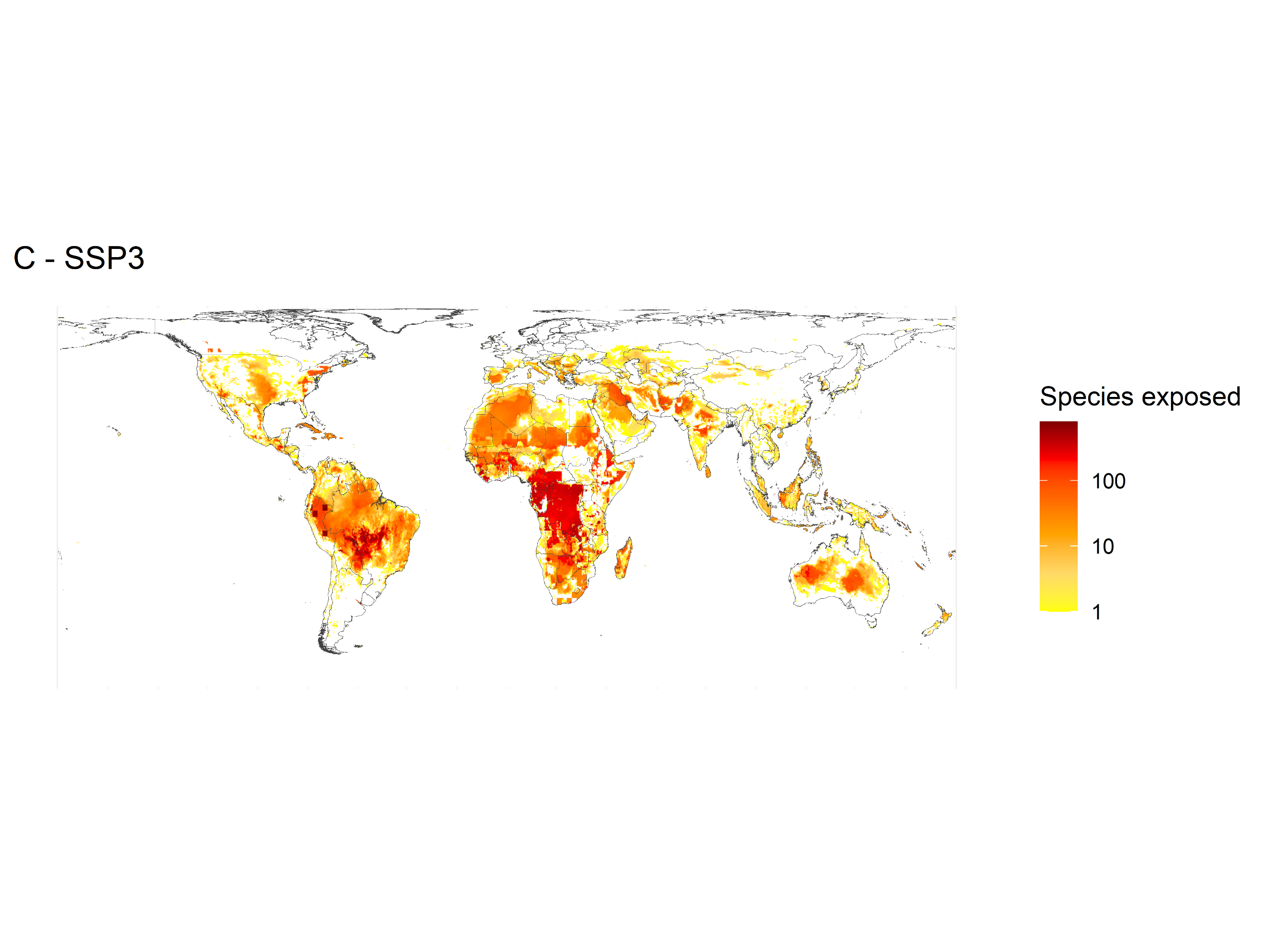

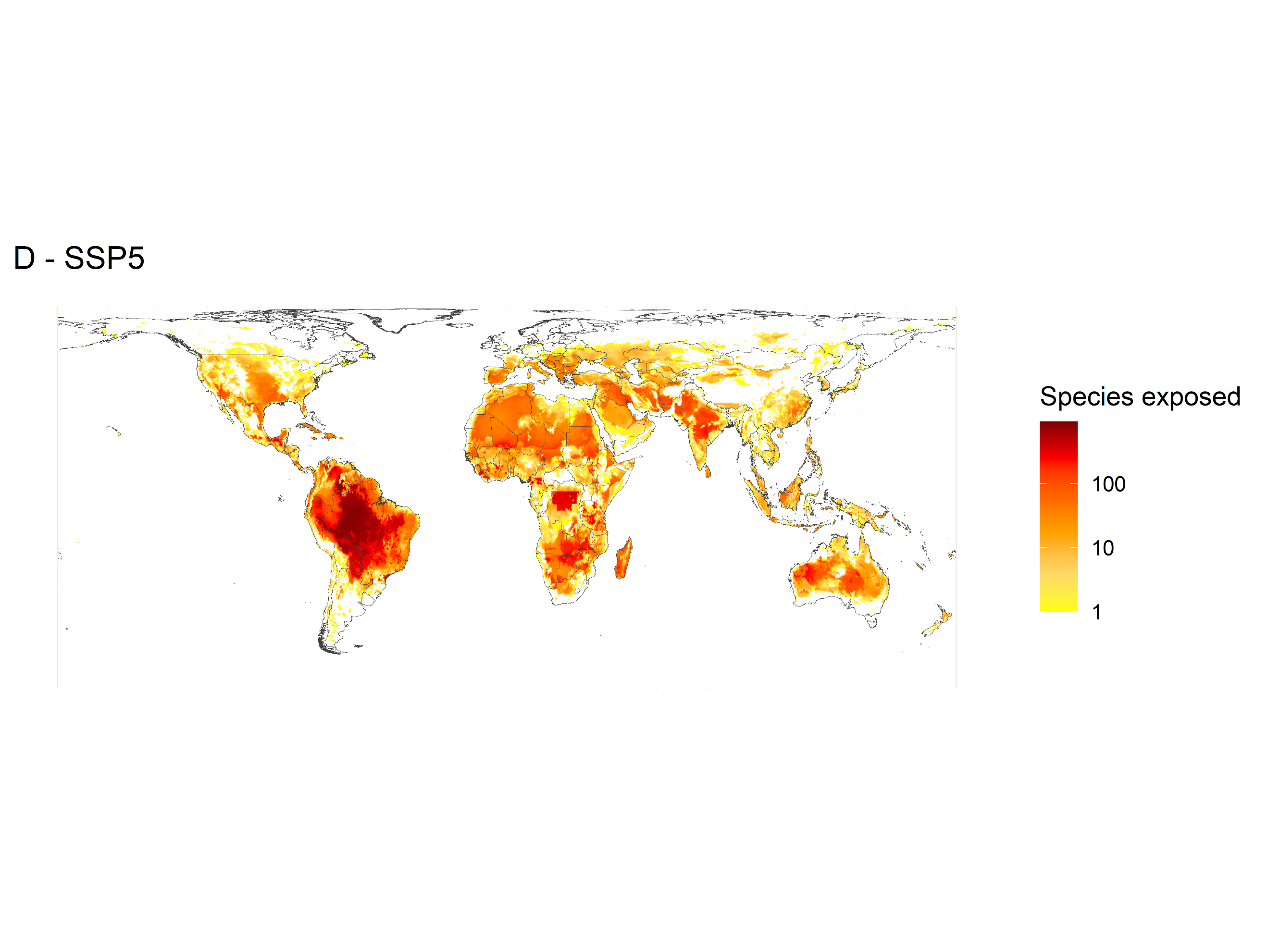

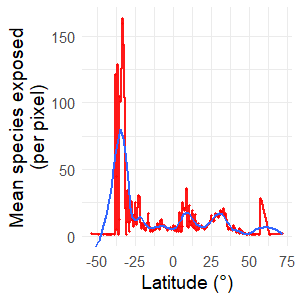

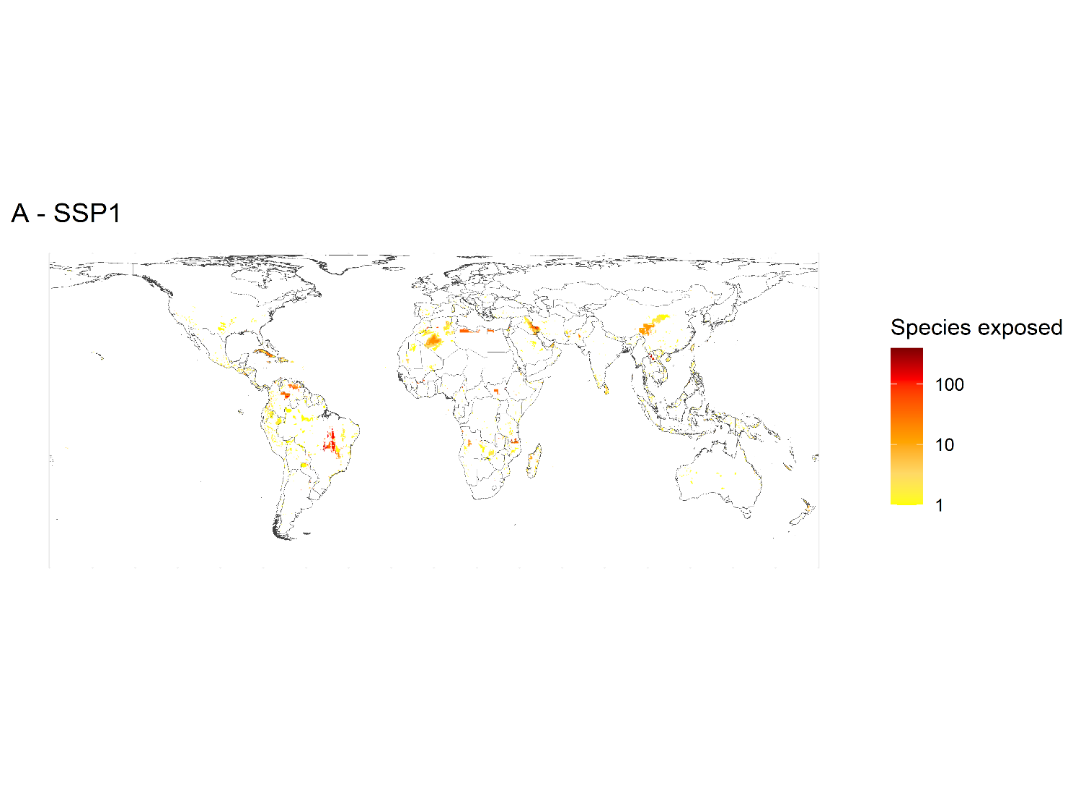

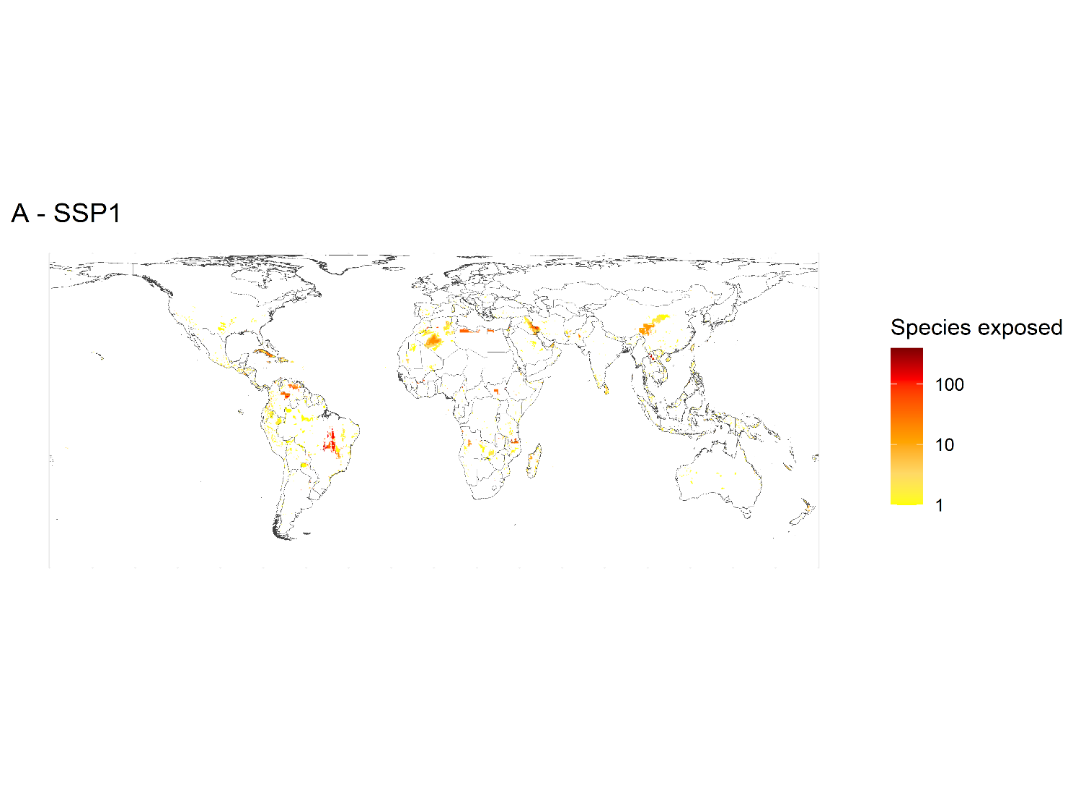

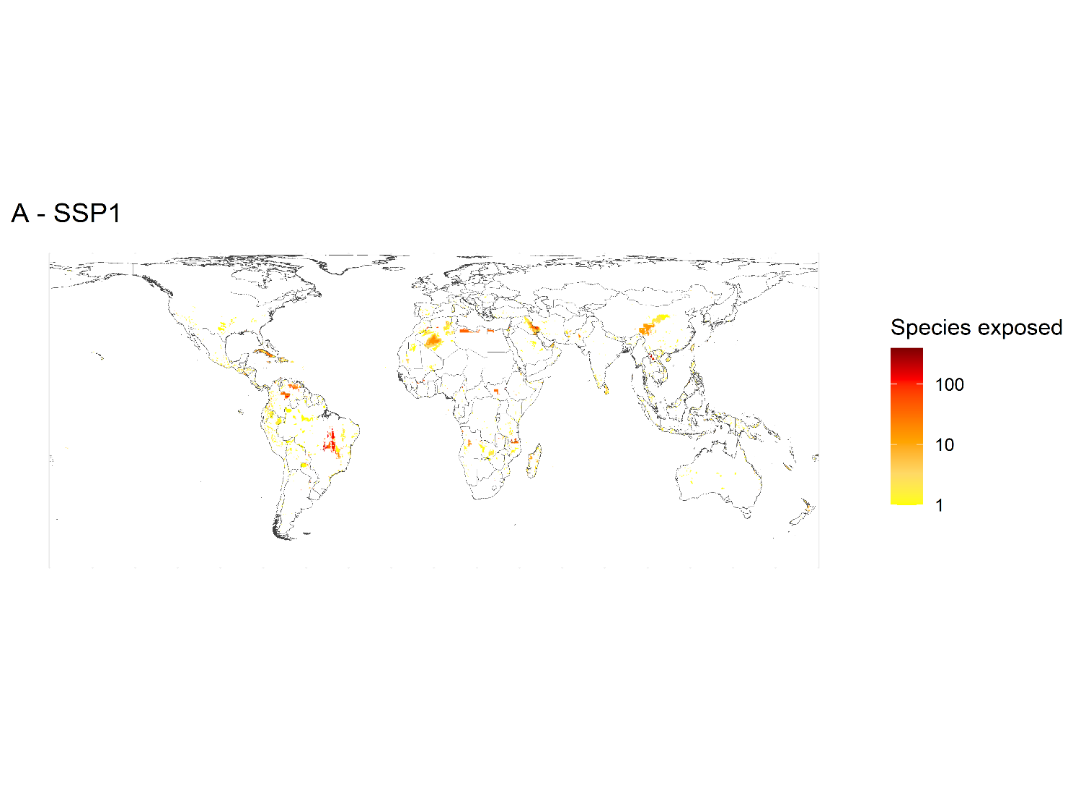

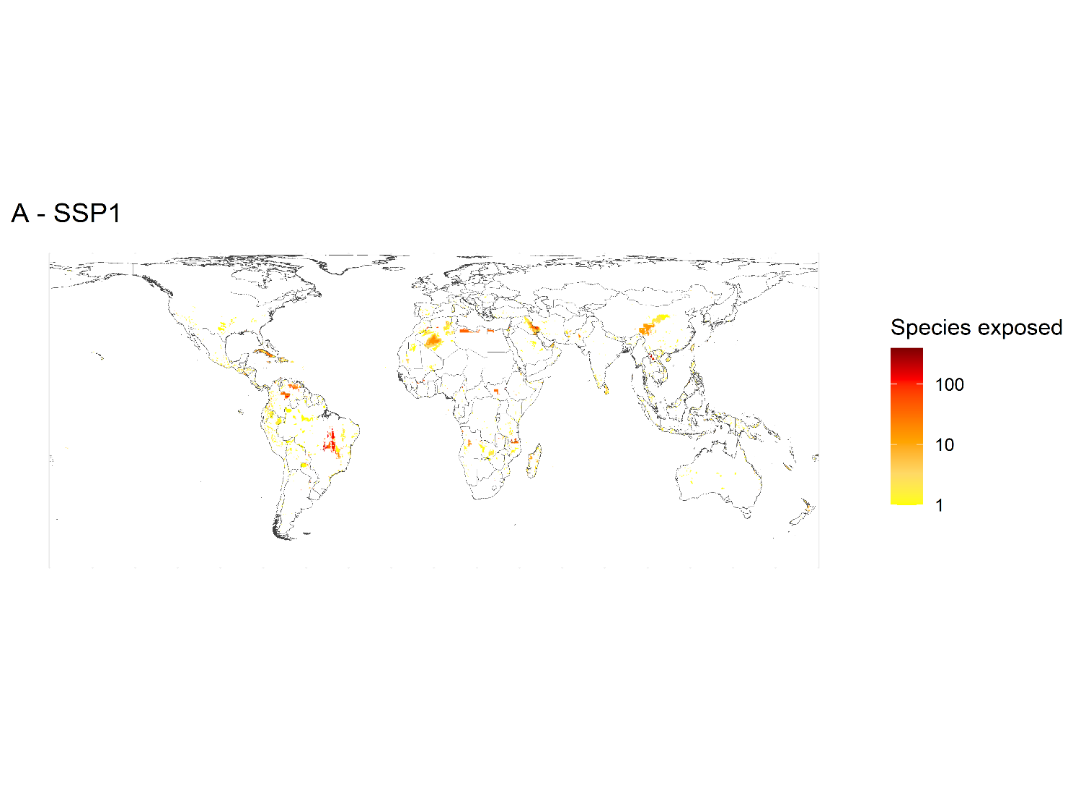

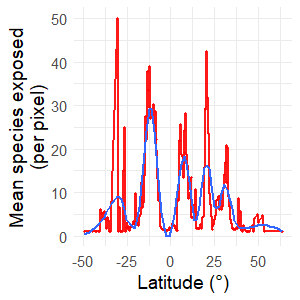

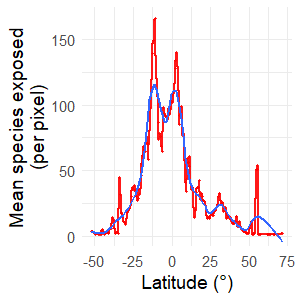

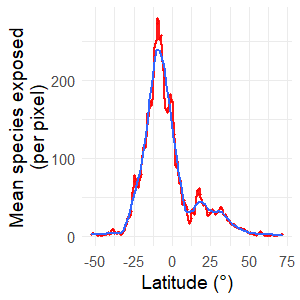

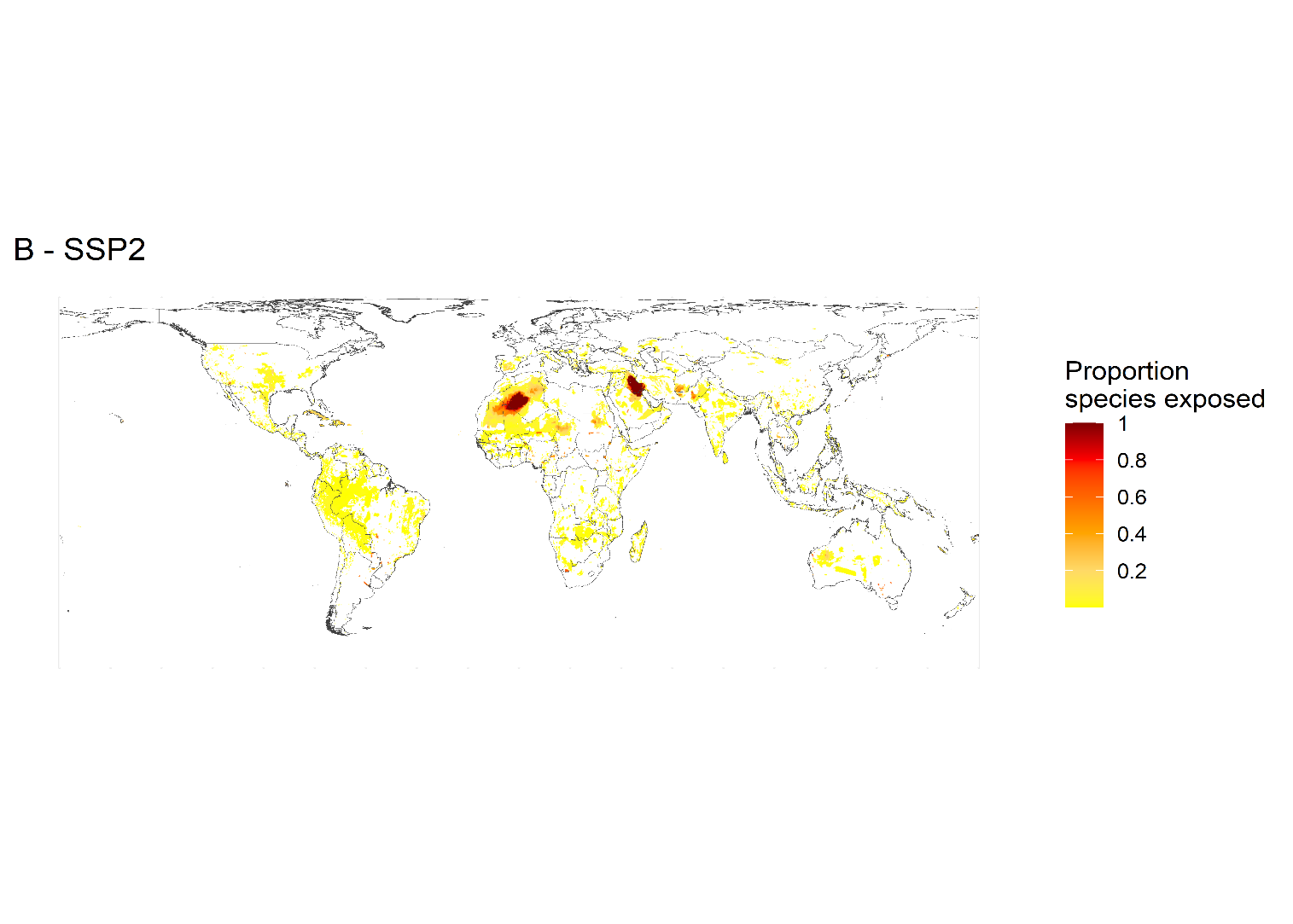

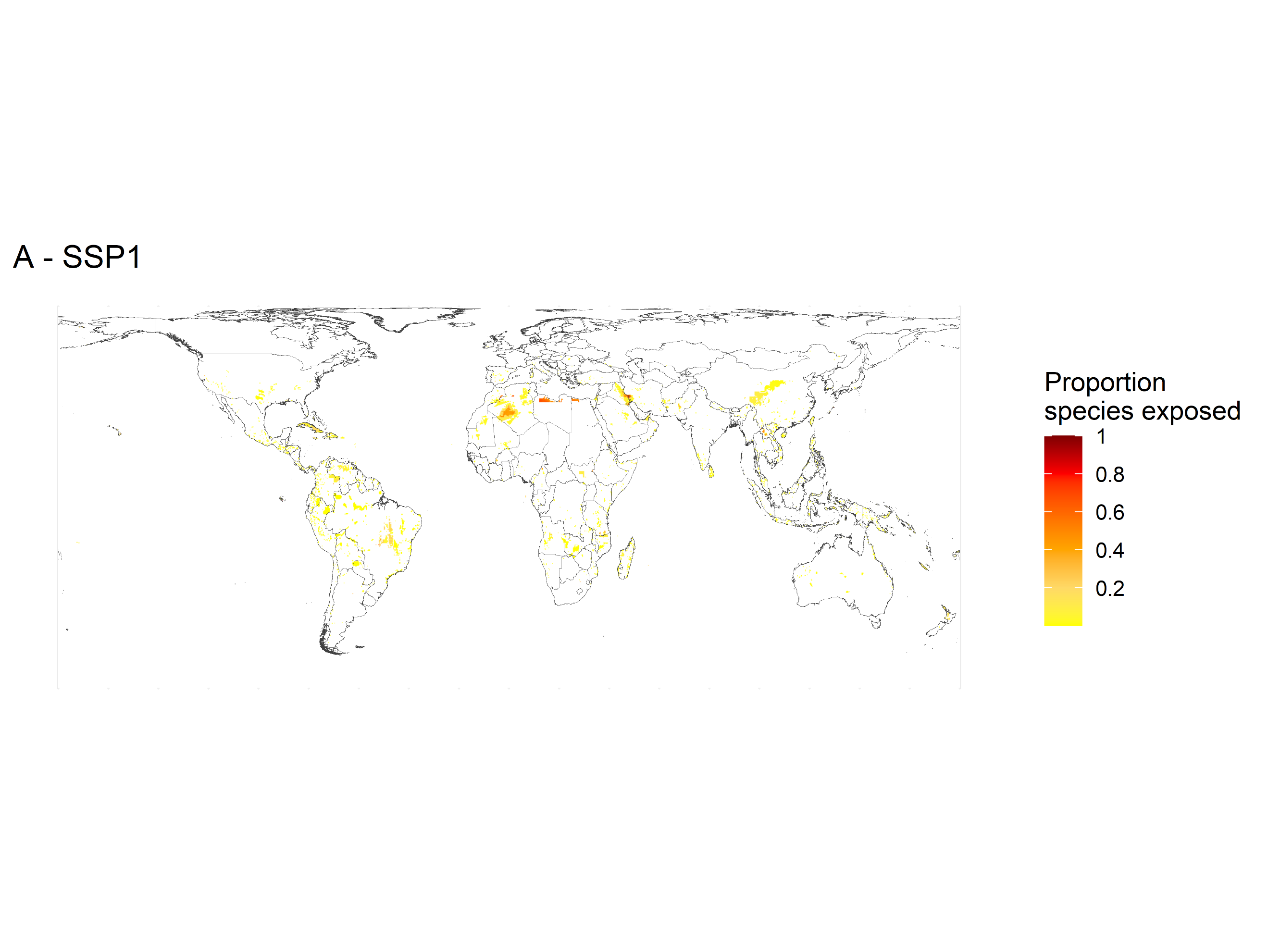

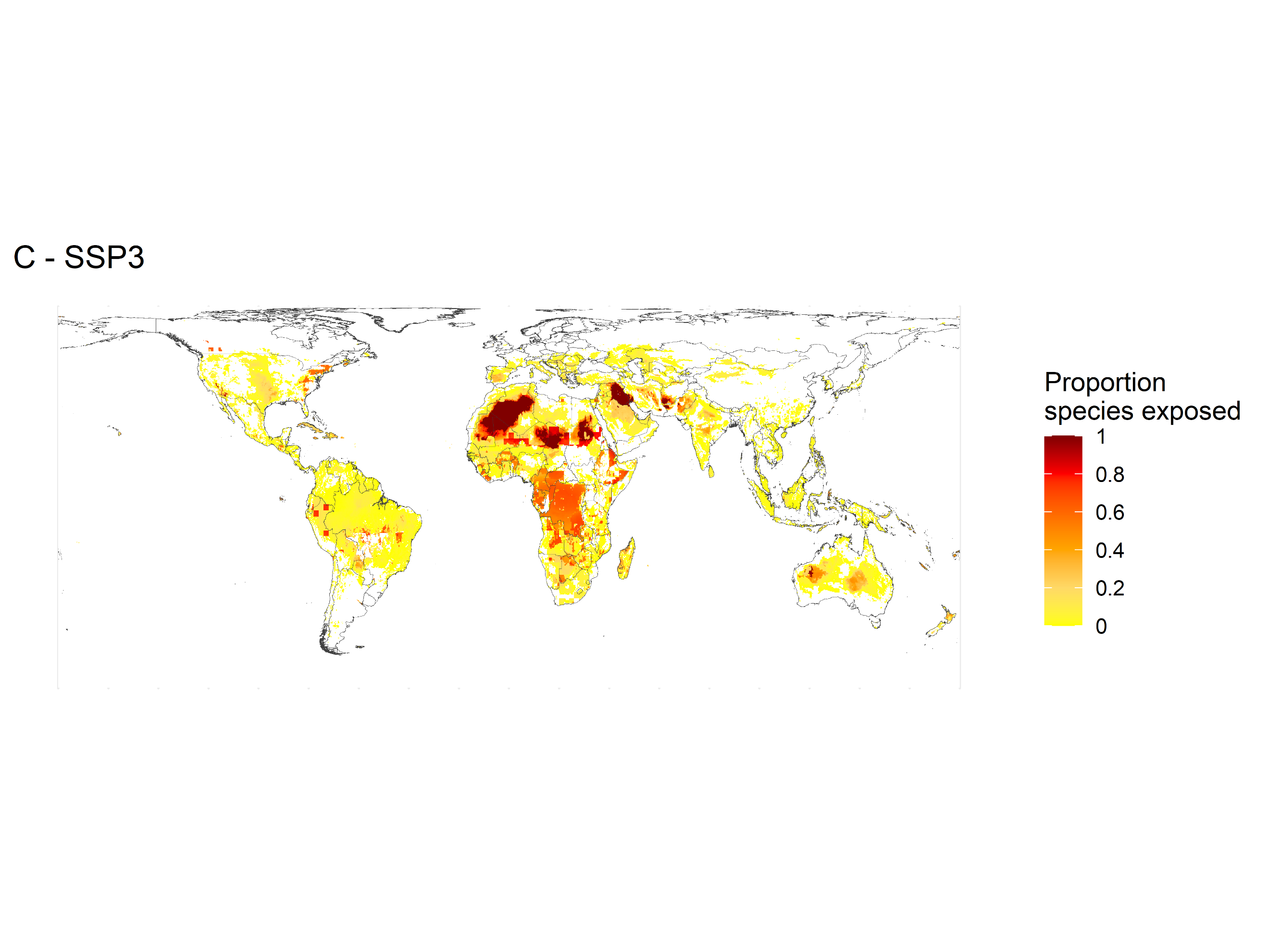

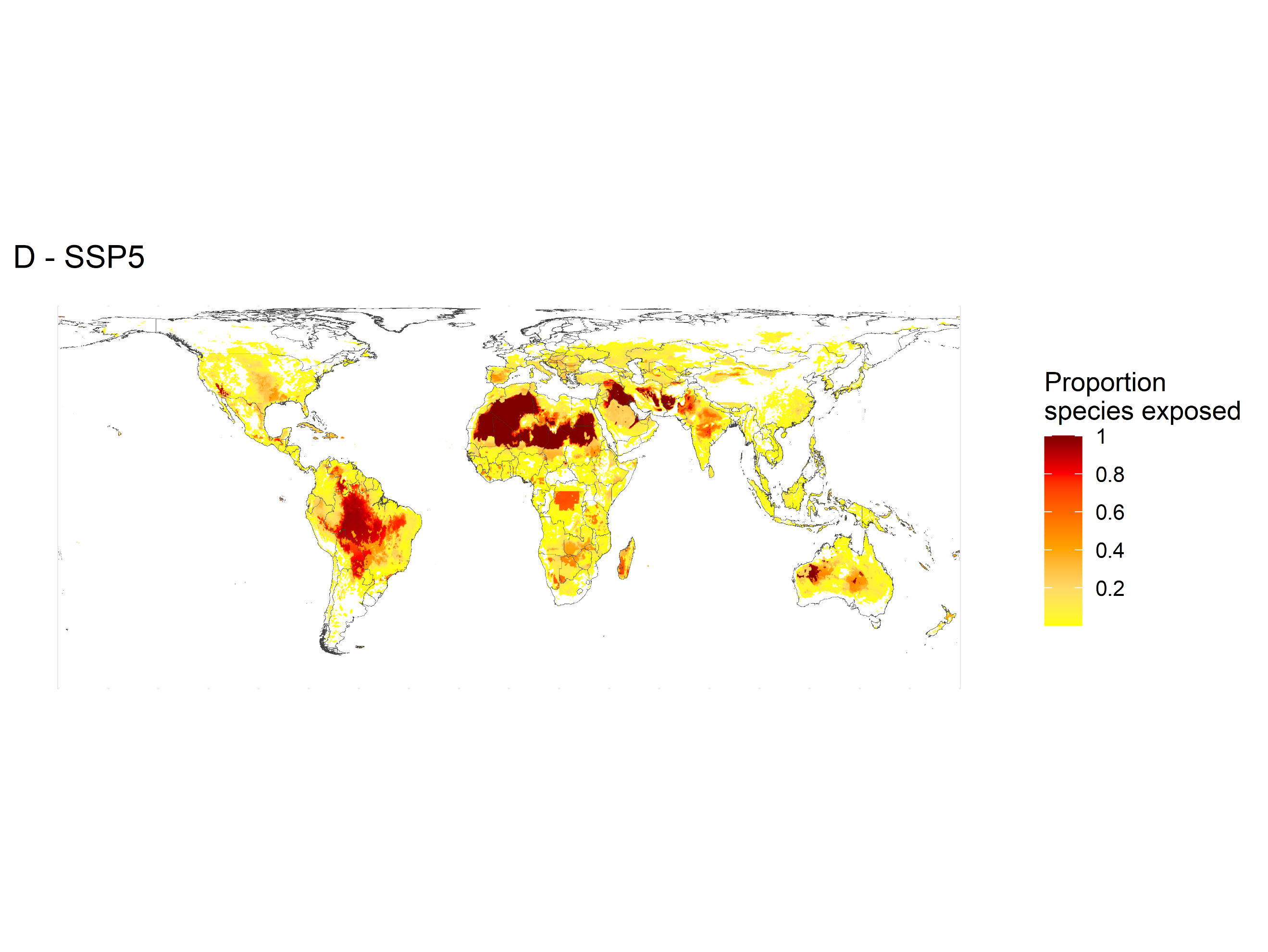


Fig. S10: The proportion of species per grid cell expected to be exposed in at least 50% of their suitable area.


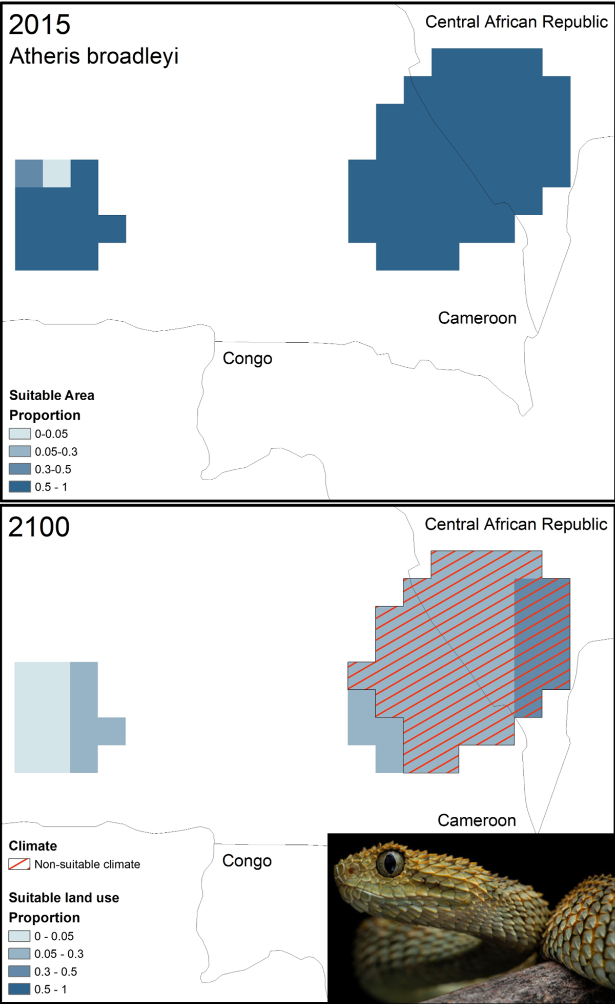


Fig. S11: *Atheris broadleyi* Suitable Area in 2015 and in 2100. An example of a species losing suitable area due to climate extrees in one part of its range and to land use changes in another part amounting to a greater potential loss considering both factors combined.

Table S1: Conversion table for all IUCN habitat types into the following five habitat classes: forest, non-forest, agriculture, managed, and urban.

| IUCN_1 | IUCN_1_Name | IUCN_2 | IUCN_2_Name | LandUseCat |
| --- | --- | --- | --- | --- |
| 1 | Forest | 1.1 | Boreal Forest | Forest |
| 1 | Forest | 1.2 | Subarctic Forest | Forest |
| 1 | Forest | 1.3 | Subantarctic Forest | Forest |
| 1 | Forest | 1.4 | Temperate Forest | Forest |
| 1 | Forest | 1.5 | Subtropical/Tropical Dry Forest | Forest |
| 1 | Forest | 1.6 | Subtropical/Tropical Moist Lowland Forest | Forest |
| 1 | Forest | 1.7 | Subtropical/Tropical Mangrove Forest Vegetation Above | Forest |
| 1 | Forest | 1.8 | Subtropical/Tropical Swamp Forest | Forest |
| 1 | Forest | 1.9 | Subtropical/Tropical Moist Montane Forest | Forest |
| 2 | Savanna | 2.1 | Dry Savanna | Non-Forest |
| 2 | Savanna | 2.2 | Moist Savanna | Non-Forest |
| 3 | Shrubland | 3.1 | Subarctic Shrubland | Non-Forest |
| 3 | Shrubland | 3.2 | Subantarctic Shrubland | Non-Forest |
| 3 | Shrubland | 3.3 | Boreal Shrubland | Non-Forest |
| 3 | Shrubland | 3.4 | Temperate Shrubland | Non-Forest |
| 3 | Shrubland | 3.5 | Subtropical/Tropical Dry Shrubland | Non-Forest |
| 3 | Shrubland | 3.6 | Subtropical/Tropical Moist Shrubland | Non-Forest |
| 3 | Shrubland | 3.7 | Subtropical/Tropical High Altitude Shrubland | Non-Forest |
| 3 | Shrubland | 3.8 | Mediterranean-type Shrubby Vegetation | Non-Forest |
| 4 | Grassland | 4.1 | Tundra | Non-Forest |
| 4 | Grassland | 4.2 | Subarctic Grassland | Non-Forest |
| 4 | Grassland | 4.3 | Subantarctic Grassland | Non-Forest |
| 4 | Grassland | 4.4 | Temperate Grassland | Non-Forest |
| 4 | Grassland | 4.5 | Subtropical/Tropical Dry Lowland Grassland | Non-Forest |
| 4 | Grassland | 4.6 | Subtropical/Tropical Seasonally Wet/Flooded Lowland G | Non-Forest |
| 4 | Grassland | 4.7 | Subtropical/Tropical High Altitude Grassland | Non-Forest |
| 5 | Wetlands (inland) | 5.1 | Permanent Rivers, Streams, Creeks [includes waterfall | Non-Forest |
| 5 | Wetlands (inland) | 5.1 | Tundra Wetlands [includes pools and temporary waters | Non-Forest |
| 5 | Wetlands (inland) | 5.11 | Alpine Wetlands [includes temporary waters from snow | Non-Forest |
| 5 | Wetlands (inland) | 5.12 | Geothermal Wetlands | Non-Forest |
| 5 | Wetlands (inland) | 5.13 | Permanent Inland Deltas | Non-Forest |
| 5 | Wetlands (inland) | 5.14 | Permanent Saline, Brackish or Alkaline Lakes | Non-Forest |
| 5 | Wetlands (inland) | 5.15 | Seasonal/Intermittent Saline, Brackish or Alkaline L | Non-Forest |
| 5 | Wetlands (inland) | 5.16 | Permanent Saline, Brackish or Alkaline Marshes/Pools | Non-Forest |
| 5 | Wetlands (inland) | 5.17 | Seasonal/Intermittent Saline, Brackish or Alkaline M | Non-Forest |
| 5 | Wetlands (inland) | 5.18 | Karst and Other Subterranean Inland Aquatic Systems | Non-Forest |
| 5 | Wetlands (inland) | 5.2 | Seasonal/Intermittent/Irregular Rivers, Streams, Cree | Non-Forest |
| 5 | Wetlands (inland) | 5.3 | Shrub Dominated Wetlands | Non-Forest |
| 5 | Wetlands (inland) | 5.4 | Bogs, Marshes, Swamps, Fens, Peatlands [generally ove | Non-Forest |
| 5 | Wetlands (inland) | 5.5 | Permanent Freshwater Lakes [over 8 ha] | Non-Forest |
| 5 | Wetlands (inland) | 5.6 | Seasonal/Intermittent Freshwater Lakes [over 8 ha] | Non-Forest |
| 5 | Wetlands (inland) | 5.7 | Permanent Freshwater Marshes/Pools [under 8 ha] | Non-Forest |
| 5 | Wetlands (inland) | 5.8 | Seasonal/Intermittent Freshwater Marshes/Pools [under | Non-Forest |
| 5 | Wetlands (inland) | 5.9 | Freshwater Springs and Oases | Non-Forest |
| 6 | Rocky Areas | 6 | areas (eg. inland cliffs, mountain peaks) | Non-Forest |
| 7 | Caves and Subterranean Habitats (nonaquatic) | 7.1 | Caves | Forest |
| 7 | Caves and Subterranean Habitats (nonaquatic) | 7.2 | Other Subterranean Habitats | Forest |
| 8 | Desert | 8.1 | Hot | Non-Forest |
| 8 | Desert | 8.2 | Temperate | Non-Forest |
| 8 | Desert | 8.3 | Cold | Non-Forest |
| 9 | Marine Neritic | 9.1 | Marine Neritic - Estuaries | NC |
| 9 | Marine Neritic | 9.1 | Marine Neritic - Pelagic | NC |
| 9 | Marine Neritic | 9.2 | Marine Neritic - Subtidal Rock and Rocky Reefs | NC |
| 9 | Marine Neritic | 9.3 | Marine Neritic - Subtidal Loose Rock/pebble/gravel | NC |
| 9 | Marine Neritic | 9.4 | Marine Neritic - Subtidal Sandy | NC |
| 9 | Marine Neritic | 9.5 | Marine Neritic - Subtidal Sandy-Mud | NC |
| 9 | Marine Neritic | 9.6 | Marine Neritic - Subtidal Muddy | NC |
| 9 | Marine Neritic | 9.7 | Marine Neritic - Macroalgal/Kelp | NC |
| 9 | Marine Neritic | 9.8 | Marine Neritic - Coral Reef | NC |
| 9 | Marine Neritic | 9.9 | Marine Neritic - Seagrass (Submerged) | NC |
| 10 | Marine Oceanic | 10.1 | Marine Oceanic - Epipelagic (0-200m) | NC |
| 10 | Marine Oceanic | 10.2 | Marine Oceanic - Mesopelagic (200-1000m) | NC |
| 10 | Marine Oceanic | 10.3 | Bathypelagic (1,000–4,000 m) | NC |
| 10 | Marine Oceanic | 10.4 | Abyssopelagic (4,000–6,000 m) | NC |
| 12 | Marine Intertidal | 12.1 | Marine Intertidal - Rocky Shoreline | NC |
| 12 | Marine Intertidal | 12.2 | Marine Intertidal - Sandy Shoreline and/or Beaches, Sand Bars, Spits, Etc | NC |
| 12 | Marine Intertidal | 12.3 | Marine Intertidal - Shingle and/or Pebble Shoreline and/or Beaches | NC |
| 12 | Marine Intertidal | 12.4 | Marine Intertidal - Mud Flats and Salt Flats | NC |
| 12 | Marine Intertidal | 12.5 | Marine Intertidal - Salt Marshes (Emergent Grasses) | NC |
| 12 | Marine Intertidal | 12.6 | Marine Intertidal - Tidepools | NC |
| 12 | Marine Intertidal | 12.7 | Marine Intertidal - Mangrove Submerged Roots | NC |
| 13 | Marine Coastal/supratidal | 13.1 | Marine Coastal/Supratidal - Sea Cliffs and Rocky Offshore Islands | NC |
| 13 | Marine Coastal/supratidal | 13.2 | Marine Coastal/supratidal - Coastal Caves/Karst | NC |
| 13 | Marine Coastal/supratidal | 13.3 | Marine Coastal/Supratidal - Coastal Sand Dunes | NC |
| 13 | Marine Coastal/supratidal | 13.4 | Marine Coastal/Supratidal - Coastal Brackish/Saline Lagoons/Marine Lakes | NC |
| 13 | Marine Coastal/supratidal | 13.5 | Marine Coastal/Supratidal - Coastal Freshwater Lakes | NC |
| 14 | Artificial – Terrestrial | 14.1 | Arable Land | Crop |
| 14 | Artificial – Terrestrial | 14.2 | Pastureland | Managed land |
| 14 | Artificial – Terrestrial | 14.3 | Plantations | Crop |
| 14 | Artificial – Terrestrial | 14.4 | Rural Gardens | Urban |
| 14 | Artificial – Terrestrial | 14.5 | Urban Areas | Urban |
| 14 | Artificial – Terrestrial | 14.6 | Subtropical/Tropical Heavily Degraded Former Forest | Managed land |
| 15 | Artificial/Aquatic | 15.1 | Water Storage Areas [over 8 ha] | Managed land |
| 15 | Artificial/Aquatic | 15.1 | Karst and Other Subterranean Hydrological Systems [human-made] | Managed land |
| 15 | Artificial/Aquatic | 15.11 | Marine Anthropogenic Structures | NC |
| 15 | Artificial/Aquatic | 15.12 | Mariculture Cages | NC |
| 15 | Artificial/Aquatic | 15.13 | Mari/Brackish-culture Ponds | Managed land |
| 15 | Artificial/Aquatic | 15.2 | Ponds [below 8 ha] | Managed land |
| 15 | Artificial/Aquatic | 15.3 | Aquaculture Ponds | Crop |
| 15 | Artificial/Aquatic | 15.4 | Salt Exploitation Sites | Managed land |
| 15 | Artificial/Aquatic | 15.5 | Excavations (open) | Managed land |
| 15 | Artificial/Aquatic | 15.6 | Wastewater Treatment Areas | Managed land |
| 15 | Artificial/Aquatic | 15.7 | Irrigated Land [includes irrigation channels] | Crop |
| 15 | Artificial/Aquatic | 15.8 | Seasonally Flooded Agricultural Land | Crop |
| 15 | Artificial/Aquatic | 15.9 | Canals and Drainage Channels, Ditches | Managed land |
| 16 | Introduced Vegetation | 16 |  | NC |
| 17 | Other | 17 |  | NC |
| 18 | Unknown | 18 |  | NC |
| 9.8 | Marine Neritic - Coral Reef | 9.8.1 | Outer Reef Channel | NC |
| 9.8 | Marine Neritic - Coral Reef | 9.8.2 | Back Slope | NC |
| 9.8 | Marine Neritic - Coral Reef | 9.8.3 | Foreslope (Outer Reef Slope) | NC |
| 9.8 | Marine Neritic - Coral Reef | 9.8.4 | Lagoon | NC |
| 9.8 | Marine Neritic - Coral Reef | 9.8.5 | Inter-Reef Soft Substrate | NC |
| 9.8 | Marine Neritic - Coral Reef | 9.8.6 | Inter-Reef Rubble Substrate | NC |

Table S2: Conversion table for the LUH2 habitat categories into the following five habitat classes: forest, non-forest, agriculture, managed, and urban.

| Generalized land cover class | Land-use Harmonization (LUH2) Land-use class |
| --- | --- |
| Forest | 1. Primf: forested primary land |
| Forest | 2. Secdf: potentially forested secondary land |
| Non-Forest | 3. Primn: non-forested primary land |
| Non-Forest | 4. Secdn: potentially non-forested secondary land |
| Managed land | 5. Pastr: managed pasture |
| Managed land | 6. Range: rangeland |
| Urban | 7. Urban: urban land |
| Crop | 8. C3ann: C3 annual crops |
| Crop | 9. C3per: C3 perennial crops |
| Crop | 10. C4ann: C4 annual crops |
| Crop | 11. C4per: C4 perennial crops |
| Crop | 12. C3nfx: C3 nitrogen-fixi |

Table S3: We ran a zero-one inflated beta model assessing the effects of range size, SSP scenario, taxonomic group, and IUCN status on the remaining suitable area in 2100. A likelihood ratio test indicated that all predictors significantly contributed to explaining variation in the remaining suitable area, with SSP and range size having the largest effects.

| Term | df | LRT | ΔAIC | p-value |
| --- | --- | --- | --- | --- |
| Range size | 1 | 4348.8 | 4347 | <0.001 |
| SSP | 3 | 20674.9 | 20669 | <0.001 |
| Taxa | 3 | 319.4 | 314 | <0.001 |
| IUCN status | 5 | 232.1 | 223 | <0.001 |

Table S4: Decadal Suitable Area losses due to climate extremes and land use changes separately and combined for all species and each taxonomic group.

| All taxa | SSP1 |  |  | SSP2 |  |  | SSP3 |  |  | SSP5 |  |  |
| --- | --- | --- | --- | --- | --- | --- | --- | --- | --- | --- | --- | --- |
| Year | Climate&Land | Climate | Land | Climate&Land | Climate | Land | Climate&Land | Climate | Land | Climate&Land | Climate | Land |
| 2015 | 100 | 100 | 100 | 100 | 100 | 100 | 100 | 100 | 100 | 100 | 100 | 100 |
| 2020 | 98.521 | 98.01371 | 100.5191 | 98.75889 | 99.57307 | 99.17595 | 97.07754 | 99.16443 | 97.91051 | 99.20256 | 99.74911 | 99.4513 |
| 2030 | 95.42472 | 94.07903 | 101.3991 | 96.06845 | 98.46643 | 97.53551 | 92.58434 | 97.38096 | 95.12587 | 94.63763 | 97.73372 | 96.88066 |
| 2040 | 95.32893 | 94.08771 | 101.3085 | 92.71399 | 96.07058 | 96.4879 | 88.67409 | 94.81401 | 93.60486 | 89.09397 | 92.78292 | 95.90638 |
| 2050 | 94.35255 | 92.61383 | 101.8184 | 89.22525 | 92.83099 | 96.03982 | 84.94886 | 92.15414 | 92.32895 | 82.39253 | 86.81697 | 94.92151 |
| 2060 | 92.80397 | 90.71625 | 102.1956 | 86.51252 | 89.78433 | 96.23837 | 78.01757 | 85.8607 | 91.10535 | 80.74029 | 85.12489 | 94.92835 |
| 2070 | 92.48788 | 90.26559 | 102.3655 | 85.23273 | 88.13312 | 96.5173 | 73.07891 | 81.07222 | 90.12985 | 67.83922 | 71.44091 | 94.98451 |
| 2080 | 92.14602 | 90.41711 | 101.7948 | 84.08109 | 86.78479 | 96.72224 | 66.88197 | 74.63677 | 89.45847 | 65.64536 | 68.78557 | 95.22127 |
| 2090 | 91.11668 | 89.89955 | 101.2361 | 80.91443 | 83.15058 | 97.04751 | 66.23427 | 74.53693 | 88.93561 | 58.8707 | 60.85815 | 96.49885 |
| 2100 | 89.35091 | 87.41704 | 102.0713 | 77.69311 | 79.20624 | 97.81227 | 53.49975 | 60.08563 | 88.4346 | 48.07929 | 49.10663 | 97.57101 |
|  |  |  |  |  |  |  |  |  |  |  |  |  |
| Amphibians | SSP1 |  |  | SSP2 |  |  | SSP3 |  |  | SSP5 |  |  |
| Year | Climate&Land | Climate | Land | Climate&Land | Climate | Land | Climate&Land | Climate | Land | Climate&Land | Climate | Land |
| 2015 | 100 | 100 | 100 | 100 | 100 | 100 | 100 | 100 | 100 | 100 | 100 | 100 |
| 2020 | 97.90159 | 97.63118 | 100.3033 | 98.4869 | 99.52375 | 98.94616 | 96.90359 | 98.98003 | 97.91413 | 98.30706 | 99.72838 | 98.57563 |
| 2030 | 91.0773 | 90.30197 | 100.8245 | 94.77325 | 97.63769 | 97.00359 | 91.33632 | 96.2246 | 94.90183 | 91.32531 | 96.7588 | 94.46588 |
| 2040 | 90.45167 | 89.94834 | 100.5546 | 89.92048 | 93.95516 | 95.64325 | 86.37362 | 92.50422 | 93.25387 | 81.80417 | 87.3583 | 93.07432 |
| 2050 | 88.36703 | 87.60329 | 100.8687 | 84.03786 | 88.10268 | 95.12724 | 80.52543 | 87.65608 | 91.80305 | 71.00525 | 77.30014 | 91.85846 |
| 2060 | 84.49047 | 83.76465 | 100.8799 | 79.50021 | 83.07096 | 95.51024 | 68.56264 | 75.80623 | 90.51246 | 68.94148 | 75.12416 | 91.89241 |
| 2070 | 83.39954 | 82.85929 | 100.7466 | 76.99005 | 79.95831 | 95.85298 | 61.38255 | 68.3731 | 89.3433 | 50.63822 | 55.15611 | 91.93585 |
| 2080 | 82.80914 | 82.95655 | 99.96023 | 74.60327 | 77.46836 | 95.93673 | 52.64592 | 58.95547 | 88.50541 | 48.11739 | 51.87661 | 92.05598 |
| 2090 | 80.89652 | 81.68457 | 99.14697 | 68.47841 | 71.08329 | 96.02962 | 52.71259 | 59.70802 | 87.85577 | 40.74953 | 43.38236 | 93.10894 |
| 2100 | 76.66526 | 76.74056 | 100.0274 | 62.02576 | 64.06624 | 96.61978 | 36.26617 | 40.71774 | 87.14485 | 28.78448 | 30.21067 | 94.24395 |
|  |  |  |  |  |  |  |  |  |  |  |  |  |
| Birds | SSP1 |  |  | SSP2 |  |  | SSP3 |  |  | SSP5 |  |  |
| Year | Climate&Land | Climate | Land | Climate&Land | Climate | Land | Climate&Land | Climate | Land | Climate&Land | Climate | Land |
| 2015 | 100 | 100 | 100 | 100 | 100 | 100 | 100 | 100 | 100 | 100 | 100 | 100 |
| 2020 | 99.19424 | 98.76949 | 100.4101 | 99.20411 | 99.80254 | 99.39542 | 97.88607 | 99.58586 | 98.29759 | 99.54202 | 99.88048 | 99.65881 |
| 2030 | 98.5824 | 97.49378 | 101.0543 | 97.53024 | 99.27999 | 98.21163 | 94.68685 | 98.64955 | 95.99347 | 96.49932 | 98.86703 | 97.61388 |
| 2040 | 98.68892 | 97.64655 | 101.0207 | 95.71315 | 98.25684 | 97.41352 | 91.98054 | 97.23224 | 94.63616 | 93.99974 | 96.97412 | 96.92308 |
| 2050 | 98.59209 | 96.96967 | 101.5729 | 93.77833 | 96.65535 | 97.04622 | 89.57614 | 95.99602 | 93.40531 | 90.71835 | 94.23158 | 96.35129 |
| 2060 | 98.67777 | 96.61624 | 101.9998 | 92.37607 | 95.07529 | 97.13817 | 85.81258 | 93.21662 | 92.22095 | 89.87138 | 93.39537 | 96.37698 |
| 2070 | 98.69773 | 96.45376 | 102.1942 | 92.41785 | 94.86409 | 97.33874 | 82.82023 | 90.77136 | 91.26446 | 80.47139 | 83.60908 | 96.45318 |
| 2080 | 98.37893 | 96.57041 | 101.73 | 91.97419 | 94.36043 | 97.42448 | 78.43647 | 86.52615 | 90.68479 | 79.37517 | 82.24008 | 96.69969 |
| 2090 | 98.08965 | 96.75478 | 101.2754 | 90.20393 | 92.26467 | 97.66403 | 78.24195 | 86.84404 | 90.18872 | 72.84692 | 74.61978 | 97.85906 |
| 2100 | 98.16281 | 96.16468 | 101.9913 | 89.61927 | 91.16297 | 98.24596 | 67.75083 | 75.26295 | 89.78766 | 63.46939 | 64.31522 | 98.88958 |
|  |  |  |  |  |  |  |  |  |  |  |  |  |
| Mammals | SSP1 |  |  | SSP2 |  |  | SSP3 |  |  | SSP5 |  |  |
| Year | Climate&Land | Climate | Land | Climate&Land | Climate | Land | Climate&Land | Climate | Land | Climate&Land | Climate | Land |
| 2015 | 100 | 100 | 100 | 100 | 100 | 100 | 100 | 100 | 100 | 100 | 100 | 100 |
| 2020 | 99.47708 | 98.90119 | 100.5622 | 98.97014 | 99.7993 | 99.17069 | 97.45675 | 99.71533 | 97.74758 | 99.40538 | 99.90025 | 99.50439 |
| 2030 | 98.07302 | 96.53462 | 101.5422 | 96.64927 | 99.24637 | 97.38935 | 93.37436 | 98.59675 | 94.78312 | 95.59165 | 98.86343 | 96.74249 |
| 2040 | 98.26694 | 96.73716 | 101.5533 | 94.52776 | 98.13336 | 96.34353 | 90.42381 | 97.32377 | 93.06175 | 91.90874 | 96.19475 | 95.55006 |
| 2050 | 98.08371 | 95.87901 | 102.241 | 92.43807 | 96.43708 | 95.85977 | 87.76714 | 95.92894 | 91.6489 | 86.85906 | 92.40769 | 94.02575 |
| 2060 | 97.72233 | 94.96986 | 102.7877 | 91.12788 | 94.72812 | 96.05645 | 82.6624 | 91.7502 | 90.15679 | 85.10894 | 90.52858 | 93.98704 |
| 2070 | 97.70432 | 94.68415 | 103.0303 | 90.47567 | 93.66113 | 96.39761 | 78.12049 | 87.60805 | 88.97172 | 74.87649 | 79.60665 | 94.07714 |
| 2080 | 97.67679 | 95.06709 | 102.619 | 89.60278 | 92.4925 | 96.67269 | 72.93631 | 82.386 | 88.05581 | 72.4382 | 76.65122 | 94.35166 |
| 2090 | 96.67299 | 94.54337 | 102.1078 | 87.06372 | 89.41043 | 97.04581 | 71.97452 | 82.15899 | 87.42216 | 66.17527 | 68.85568 | 95.8266 |
| 2100 | 96.21835 | 93.21315 | 103.105 | 85.02353 | 86.40441 | 97.83768 | 60.57078 | 68.80773 | 86.80075 | 55.64368 | 57.19657 | 96.94 |
|  |  |  |  |  |  |  |  |  |  |  |  |  |
| Reptiles | SSP1 |  |  | SSP2 |  |  | SSP3 |  |  | SSP5 |  |  |
| Year | Climate&Land | Climate | Land | Climate&Land | Climate | Land | Climate&Land | Climate | Land | Climate&Land | Climate | Land |
| 2015 | 100 | 100 | 100 | 100 | 100 | 100 | 100 | 100 | 100 | 100 | 100 | 100 |
| 2020 | 97.6623 | 96.92684 | 100.7762 | 98.34168 | 99.21894 | 99.10821 | 96.08214 | 98.50286 | 97.57653 | 99.37623 | 99.52769 | 99.84687 |
| 2030 | 93.5886 | 91.6454 | 102.1271 | 95.0688 | 97.71558 | 97.273 | 90.71198 | 96.10826 | 94.53818 | 94.48446 | 96.52679 | 97.96452 |
| 2040 | 93.49153 | 91.6494 | 102.0465 | 90.38991 | 93.98894 | 96.18237 | 85.67722 | 92.35125 | 93.05178 | 87.42802 | 90.14839 | 97.12082 |
| 2050 | 91.89308 | 89.57397 | 102.5494 | 86.13098 | 89.96321 | 95.71722 | 81.43638 | 88.99214 | 91.9399 | 79.01282 | 82.36487 | 96.17656 |
| 2060 | 89.56349 | 86.82463 | 103.044 | 82.48868 | 85.97315 | 95.89662 | 73.6676 | 81.69737 | 90.88511 | 76.83519 | 80.19941 | 96.16959 |
| 2070 | 89.27671 | 86.29623 | 103.3707 | 80.28792 | 83.46931 | 96.17702 | 68.01356 | 75.9054 | 90.16047 | 62.49708 | 65.24442 | 96.19261 |
| 2080 | 88.90632 | 86.38757 | 102.7477 | 79.11034 | 81.93283 | 96.56298 | 61.10136 | 68.5476 | 89.65996 | 59.47822 | 61.81155 | 96.48336 |
| 2090 | 87.70387 | 85.66155 | 102.2361 | 76.23777 | 78.32484 | 97.12908 | 59.60358 | 67.42051 | 89.2695 | 52.56701 | 53.88958 | 97.94181 |
| 2100 | 84.95848 | 82.2329 | 103.0719 | 71.80534 | 72.96816 | 98.21157 | 46.36513 | 52.53027 | 88.88794 | 40.93149 | 41.54347 | 98.98808 |
